# The bombardment history on the lunar farside revealed by ^40^Ar/^39^Ar geochronology of Chang’e-6 impact melt rocks

**DOI:** 10.1126/sciadv.aee8718

**Published:** 2026-07-23

**Authors:** Wan-Feng Zhang, Le Zhang, Mang Lin, Fred Jourdan, Jun-Jie Wang, Yan-Qiang Zhang, Jing-You Chen, De-Wen Zheng, Peng-Li He, Fei Su, Huai-Yu He, Hua-Ning Qiu, Xiu-Juan Bai, Xiao-Ping Xia, Ming Xiao, Ying-De Jiang, Jun-Jie Li, Jia Zhang, Lie-Kun Yang, Fang-Fang Huang, Bo Wei, Jiang-Ze Wang, Qin Zhou, Yong-Hua Cao, Ze-Xian Cui, Qing Yang, Lin-Li Chen, Yi-Gang Xu

**Affiliations:** ^1^ State Key Laboratory of Deep Earth Processes and Resources, Guangzhou Institute of Geochemistry, Chinese Academy of Sciences, Guangzhou 510640, China.; ^2^ Center for Advanced Planetary Science (CAPS), Guangzhou Institute of Geochemistry, Chinese Academy of Sciences, Guangzhou 510640, China.; ^3^ Western Australian Argon Isotope Facility, John de Laeter Centre & School of Earth and Planetary Sciences, Curtin University, Perth 6845, Australia.; ^4^ Institute of Geology, China Earthquake Administration, State Key Laboratory of Earthquake Dynamics and Forecasting, Beijing 100029, China.; ^5^ Key Laboratory of Earth and Planetary Physics, Institute of Geology and Geophysics, Chinese Academy of Sciences, Beijing 100037, China.; ^6^ Key Laboratory of Tectonics and Petroleum Resources, Ministry of Education, China University of Geoscience, Wuhan 430074, China.; ^7^ College of Resources and Environment, Yangtze University, Wuhan 430100, China.; ^8^ NWU-HKU Joint Centre of Earth and Planetary Sciences, Department of Earth and Planetary Sciences, The University of Hong Kong, Hong Kong, China.; ^9^ Beijing Research Institute of Uranium Geology, Beijing 10029, China.; ^10^ State Key Laboratory of Lithospheric and Environmental Coevolution, Institute of Geology and Geophysics, Chinese Academy of Sciences, Beijing 100037, China.

## Abstract

The reconstruction of the lunar bombardment history is pivotal to understanding the evolution of Earth-Moon system. So far, it heavily depends on geochronological studies of lunar nearside samples but lacks direct sample constraints from the lunar farside. Here, we present 28 single-clast ^40^Ar/^39^Ar ages of impact melt rocks returned by the Chang’e-6 mission. They reveal impact events spanning from ∼4.33 to ∼1.13 billion years (Ga). In particular, three melt clasts date to ∼4.16 Ga, consistent with the recent dating of the Apollo basin, and only two ages fall at 4.0 to 3.7 Ga. The presence of older ages (>4.0 Ga) and lack of predominant age around ∼3.9 Ga suggest that the lunar farside bombardment was not dominated by a cataclysmic spike but by a smooth long-term decline punctuated by later impacts.

## Teaser

First lunar farside ^40^Ar/^39^Ar impact melt ages from Chang’e-6 challenge the cataclysmic LHB model.

## INTRODUCTION

In the early 1970s, ^40^Ar/^39^Ar (*1*, *2*), U-Pb, and Rb-Sr (*3*, *4*) isotopic analyses of impact melt breccias collected by Apollo 15, 16, and 17, as well as the Luna 20 mission, revealed widespread and unexpectedly consistent isotopic disturbances at approximately 3.9 Ga, suggesting that at least three and probably six of the major lunar basins formed over this period (*2*). Tera *et al.* (*4*) subsequently proposed a far-reaching hypothesis that argon and lead losses (and correlated disturbances in the Rb-Sr system) were caused by metamorphism of the lunar crust resulting from an intense pulse of asteroid and/or cometary impacts, a phenomenon they termed the “terminal lunar cataclysm.” “Late Heavy Bombardment (LHB)” was first used by Wetherill (*5*) and is considered to have notably influenced and shaped the surface history of the Earth, Moon, other terrestrial planets, and the asteroid region [e.g., (*5*–*9*)].

There is little doubt that the Moon experienced a period of heavy bombardment after its formation, leading to the creation of multiple large basins. A major unresolved question is whether there was an “LHB” defined as a single, short-lived episode [lasting about 150 to 200 million years (Myr)] of intense cratering at ∼3.9 billion years (Ga) (i.e., cataclysm LHB hypothesis) (*2*, *4*, *10*), a sawtooth-like uptick impact flux earlier than about 4.1 Ga (*11*), or a “picket fence”–like bombardment scenario characterized by multiple cataclysms since ∼4.24 Ga (*12*) or whether the bombardment was spread over a longer period as a monotonically decreasing impact flux (accretion tail) (*13*–*16*). Over the past decades, ^40^Ar/^39^Ar analyses of impact melt rocks from Apollo 15 (*17*), and Apollo 17 (*18*), and Luna 20 (*19*) have consistently yielded a range of apparent ages around 3.9 Ga, seemingly in line with earlier studies, therefore reinforcing the lunar cataclysm LHB hypothesis.

Despite supporting evidence from more geochronological [e.g., (*17*–*19*)] and dynamical [e.g., (*20*, *21*)] studies, the LHB hypothesis has remained a subject of ongoing debate since its inception [e.g., (*22*–*26*)]. Filtering of published stepwise heating ^40^Ar/^39^Ar isotopic results from 259 fragments of Apollo breccia samples based on internal statistical criteria shows that most do not define true ages, but rather error ages (*27*), and only 29 of these reported ^40^Ar/^39^Ar data represent true isotopic ages that indicate a geological event. Twenty-four of these 29 ages are in fact indistinguishable within error and define an age of 3916 ± 7 Ma, which probably represents the age of a single event, i.e., formation of the Imbrium basin (*27*). In addition, based on the compositional similarity of lunar impact samples collected from multiple Apollo landing sites, Liu *et al.* (*28*), Merle *et al.* (*29*), Mercer *et al.* (*30*), and Harrison *et al.* (*31*) concluded that these samples were derived from Imbrium and thus represent one event and not several in a short time period. Thus, previous results are suspected to be substantially affected by the Imbrium impact event at ∼3.92 Ga, the largest late basin-forming event in lunar nearside (*6*, *27*), and did not unravel the true lunar bombardment history (*32*). This potential sampling bias is further confirmed by lack of any clear peak around 3.9 Ga recorded in lunar meteorite impact melt rocks (*33*, *34*), which represent a random sampling of the lunar surface. Recent Pb-Pb and U-Pb isotopic dating on impact melt clasts from Chang’e-6 (CE-6) and NWA2995 lunar meteorites yield ages clustering between 4.16 and 4.33 Ga (*14*, *35*–*37*). U-Pb and ^40^Ar/^39^Ar dating of Vesta reveals an extended impact bombardment record that challenges the prevailing cataclysm LHB models of inner Solar System impact flux, indicating that this model needs reassessment (*38*–*40*). Together, these observations challenge the single, short-lived episode lunar cataclysm hypothesis.

At first glance, the scarcity of ^40^Ar/^39^Ar ages from impact melt rocks older than ∼4.0 Ga, including all samples from the Apollo and Luna missions (*6*, *30*, *33*, *34*), is accountable because the K-Ar system is much more sensitive to resetting or partial resetting by high-temperature event compared with the U-Pb system such as encountered during the formation of the Imbrium basin formation (*41*). The preservation of ^40^Ar/^39^Ar ages older than ∼4.0 Ga would require a given melt rock to remain isolated from significant impact events after its formation (*6*, *42*). Although older melt rocks would have been progressively affected, melt from large basins is so abundant that it becomes continuously present at the surface through repeated excavations (*32*). Thus, the ^40^Ar/^39^Ar chronometer offers a unique opportunity to look for such impact events and ultimately to reconstruct the lunar bombardment history.

On the near side, most of the bombardment history recorded by melt rocks is obscured by the abundant materials produced during or affected by the Imbrium basin formation (∼3.92 Ga), and as such, the lunar bombardment history is severely masked (*27*–*31*). This problem can be overcome by examining impact melt rocks from the lunar meteorites or samples return from lunar farside. On 25 June 2024, China’s CE-6 mission brought back the first-ever lunar farside samples (1935.3 g) from the Apollo basin within the South Pole–Aitken (SPA) basin at lunar coordinates 41.625°S and 153.978°W (*43*). The SPA and Apollo basins have diameters of ∼2500 and 492 km, respectively (*44*, *45*), representing two key huge impact events during the lunar basin–forming era (>4.1Ga) (*44*, *46*). Statistics from detailed petrography and geochemical analyses reveal that the exotic materials account for 23.5 to 33.5 vol % of the CE-6 regolith, and more than 72% of the impact melt rocks in the CE-6 returned samples are locally derived (*47*). As the SPA basin is the largest and oldest recognized impact basin preserved on the Moon (*44*, *46*), the impact melt rocks recovered by the CE-6 mission represent an ideal target to look for preserved melt rocks and to test the lunar cataclysm hypothesis. In this work, we present the first ^40^Ar/^39^Ar ages obtained on impact melt rocks from the CE-6 soils. Our results reveal the presence of pre–4.0 Ga impact melt rocks and confirm that the formation age of the Apollo basin is ∼4.16 Ga, providing direct evidence of a monotonically decreasing impact flux instead of cataclysm LHB model in the early impact history of the Moon.

## METHODS

**Fig. 1.**
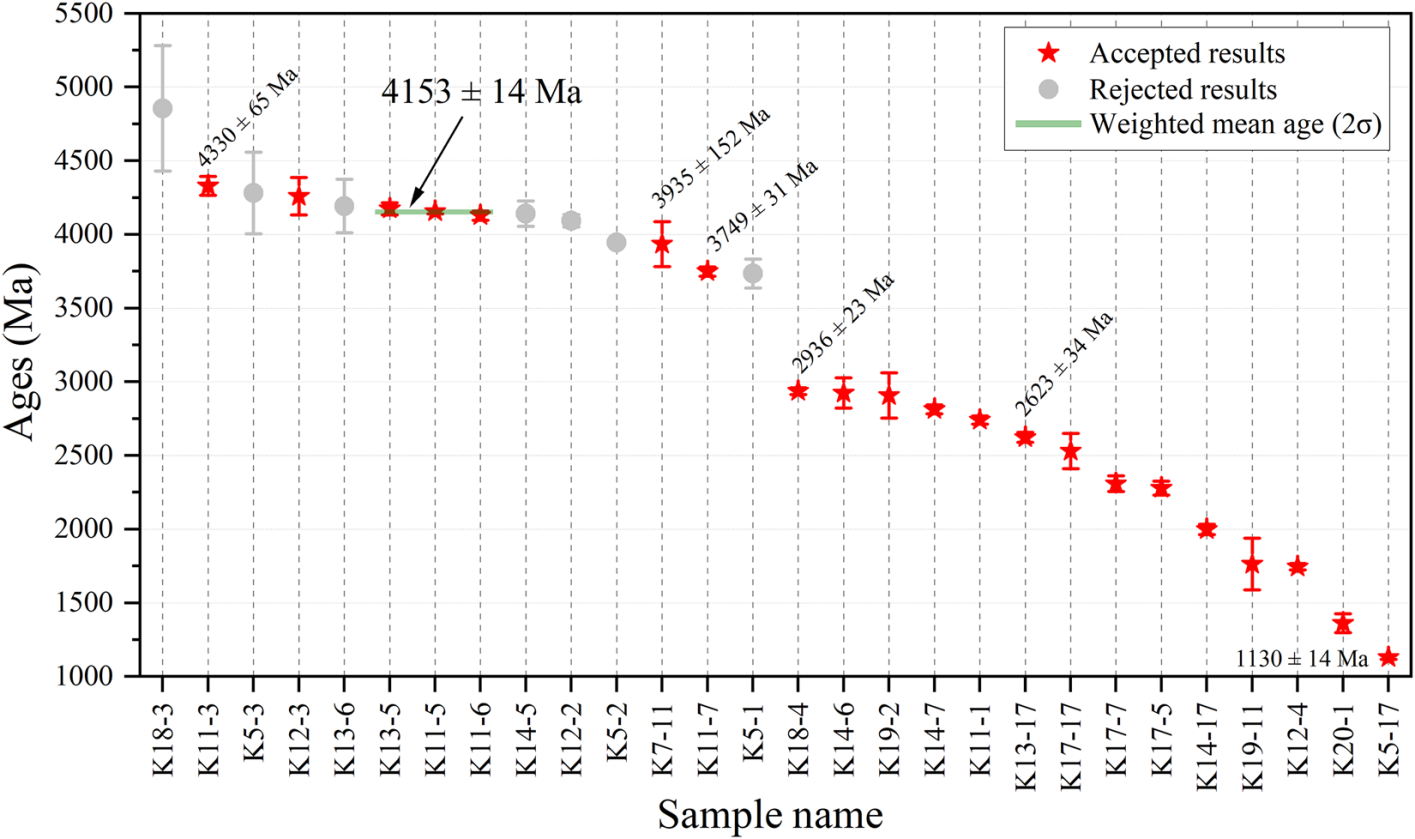
Summary of the ^40^Ar/^39^Ar ages of CE-6 impact melts obtained in this study. The gray circles represent results with either large dispersion or a ∑^39^Ar < 50% over more than three consecutive steps in their age spectra, which are deemed unreliable and excluded from further discussion. Green shading denotes the ±2σ uncertainty interval about the weighted mean age. Detailed age spectrum results and dataset for individual samples are provided in figs. S4 to S6 and table S2, respectively.

The analyzed clasts were selected from an aliquot of scooped soils from the CE-6 lunar regolith (CE6C0100YJFM003; 2 g), allocated by the China National Space Administration (CNSA). These aliquot samples contain multiple lithic fragments, including basalt, breccia, agglutinate, glasses, and leucocratic clasts (*43*), among which the impact melt clast cannot be accurately distinguished under microscope. Therefore, clasts larger than 100 μm (more than 100 particles) were first selected under a binocular microscope, then embedded in adhesive mounts (Crystalbond 509), and polished for backscattered electron (BSE) imaging for the identification of potential impact melt clast. Given that typical impact melt clasts in Apollo and Luna samples were crystalline, fine grained, microporphyritic, and micropoikilitic texture (*30*, *33*, *48*), we selected clasts with analogous characteristic for this study. In total, 28 clasts (Fig. 1) exhibiting impact melt texture and ranging in sizes from 100 to 500 μm were identified from more than 100 selected sample clasts. Each sample clast is individually packaged in aluminum foil, placed sequentially into a quartz tube, and sealed under vacuum. The samples were then irradiated for 14.5 hours in the High Flux Engineering Test Reactor in Sichuan, China. Each clast was then analyzed individually via laser stepwise heating ^40^Ar/^39^Ar dating measured with a Thermo Fisher Scientific ARGUS VI multicollection mass spectrometer coupled with a custom-made CO_2_ laser (infrared: 10.6 μm) and purification system at the State Key Laboratory of Deep Earth Processes and Resources, Guangzhou Institute of Geochemistry, Chinese Academy of Sciences (GIGCAS) (see the Supplementary Materials for more detail analytical methods).

Following laser stepwise heating ^40^Ar/^39^Ar dating analyses, each sample clast was fused into a glass bead. According to our established analytical protocol (*49*), the glass beads were retrieved from the holder, embedded in epoxy mounts (EpoThin), and polished before major and trace element analysis by laser ablation inductively coupled plasma mass spectrometry. Trace element analyses show that most of dated samples are enriched in iridium (Ir) with an average concentration of 89.2 parts per billion (ppb) and a maximum of 438.9 ppb observed in sample K14-6 (table S1), which are substantially higher than the lunar Ir concentrations of 0.1 to 10 ppb (*50*). These elevated Ir contents are consistent with the composition of a series of Apollo 16 impact melt breccias (∼7 to 35 ppb) (*51*), suggesting an impact melt origin of these fragments. These samples exhibit significant variations in major element contents (table S1): FeO (0.00 to 22.32 wt %), MgO (0.55 to 18.34 wt %), Al_2_O_3_ (6.60 to 35.99 wt %), and CaO (6.18 to 51.7 wt %). Fourteen clasts have a CaO/Al_2_O_3_ ratio below 0.75, a threshold commonly used to distinguish mare versus highland affinity (*52*). All samples but K11-7 have MgO/Al_2_O_3_ ratio below 1.25 (table S1). Although K11-7 falls within the field of volcanic glass, its elevated Ir content (19.4 ppm) indicates that it was also affected by impact. On the basis of BSE imaging and major and trace element compositions, the studied clasts are interpreted as impact melt clasts. They exhibit diverse textures, including single-mineral fragments, leucocratic material, granulite clasts, and irregular glass fragments, with very rare occurrences of glass beads and basaltic clasts. Diverse textures (fig. S1) and multiple source materials of these impact melt rocks (figs. S2 and S3) indicate that they likely resulted from different impacts or possibly multiple impact events.

## RESULTS

Following established ^40^Ar/^39^Ar data interpretation criteria, ages derived from at least three consecutive steps comprising 50 to 70% (plateau) or >70% (robust plateau) of the released ^39^Ar and that yield a probability of *P* ≥ 0.05 are considered meaningful (*27*, *53*). Trapped intercept ^40^Ar/^36^Ar ratio [(^40^Ar/^36^Ar)_tr_] were measured using the inverse isochron according to previous studies (*53*–*55*). On the basis of these criteria, 21 of 28 samples yielded meaningful plateau ages (figs. S4 to S6). These ages range from 4330 ± 65 to 1130 ± 14 Ma (Fig. 1). Sample K14-5 is distinct from the others, as it exhibits gradually increasing apparent ages, indicating partial resetting by a subsequent impact event. On a first-order approximation, the ^40^Ar/^39^Ar ages are characterized by three age ranges of ∼>4 Ga, 4 to 3 Ga, and <3 Ga, respectively (Fig. 1). When data points are concordant within a 2σ confidence interval and do not have large uncertainties (i.e., <50 Ma), such samples likely represent products of a single impact event (*27*). On the basis of these criteria, only a set of three melt clasts of ages has been grouped at 4153 ± 14 Ma (*n* = 3; *P* = 0.15; Fig. 1). Other results exhibit significant scatter beyond analytical uncertainty and are therefore interpreted as showing distinct impact ages. Uncertainties discussed in this article are all 2σ.

### Pre–4-Ga dating results

**Fig. 2.**
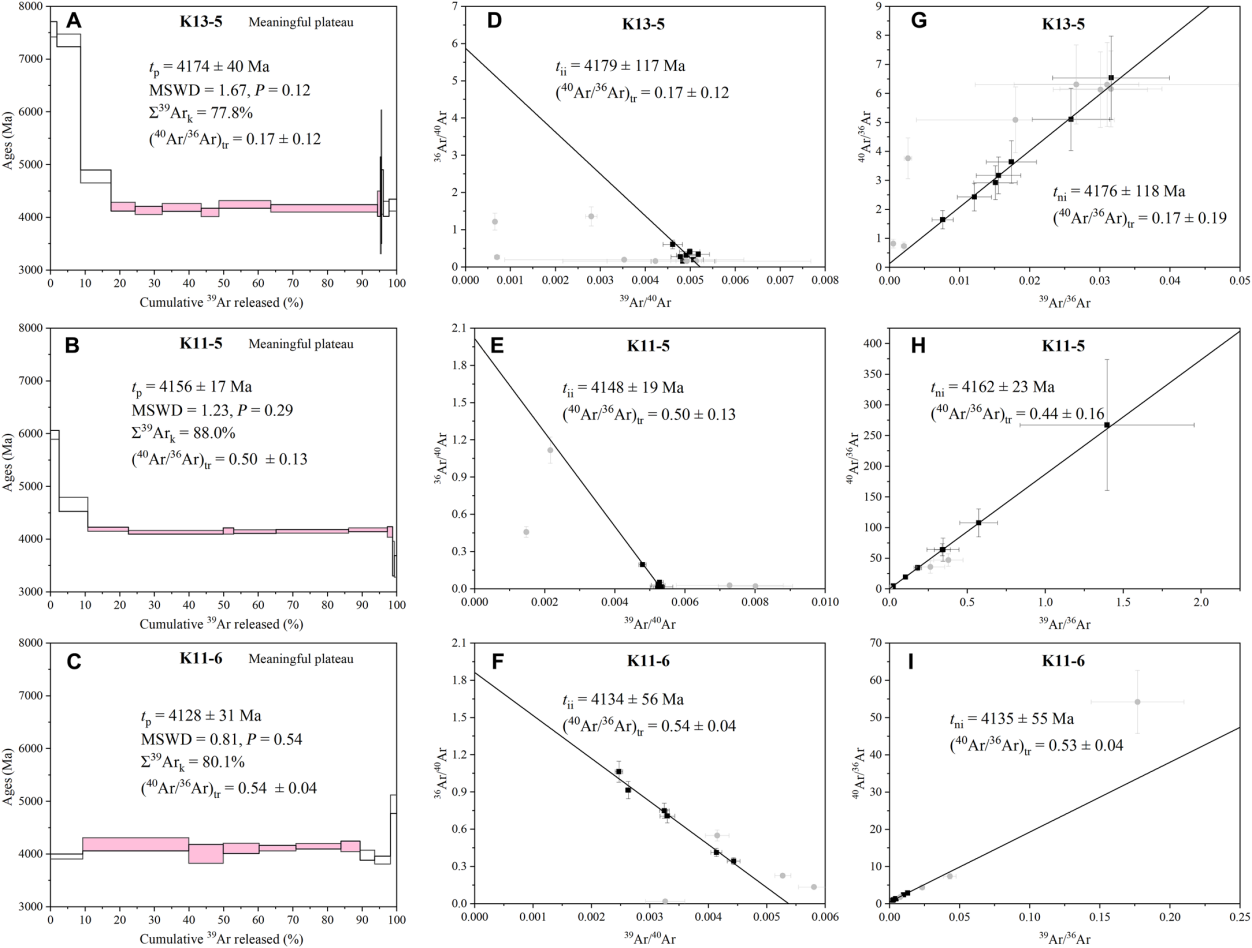
Laser stepwise ^40^Ar/^39^Ar dating results for three typical samples. Plateau age (**A** to **C**), inverse isochron (**D** to **F**), and normal isochron (**G** to **I**). Error is 2σ. Trapped intercept ^40^Ar/^36^Ar ratio [(^40^Ar/^36^Ar)_tr_] were measured using the inverse isochron according to previous studies (*53*–*55*). The color-filled steps in age spectra (A to C) were used for plateau age calculation. The black squares in isochron (D to I) represent the data used for inverse isochron regression, whereas the gray circles denote data excluded from the regression.

Ten of 28 samples (fig. S4) yield >4-Ga results, which accounts for 36% of all samples. Five of 10 samples meet the ^40^Ar/^39^Ar data interpretation criteria (cumulative released ^39^Ar > 50%, and *P* ≥ 0.05; K11-3, K12-3, K13-5, K11-5, and K11-6 in fig. S4), yielding age range from 4330 ± 65 to 4128 ± 31 Ma. Specifically, the ages of three melt clasts (K13-5, K11-5, and K11-6) are interpreted to represent a single event with a weighted mean age of 4153 ± 14 Ma (Figs. 1 and 2). Although the age obtained from sample K12-3 (4260 ± 127 Ma) is consistent within error with the weighted mean age of 4153 ± 14 Ma, the large analytical uncertainty in K12-3 precludes a robust assessment of whether these samples represent the same event. In these three samples, the initial few steps exhibit abnormal old ages, likely caused by implanted inherited parentless ^40^Ar^*^ during impact process. Their inverse isochron ages are indistinguishable from their plateau ages (Fig. 2). Other 5 of 10 samples fail to yield an age (K18-3, K5-3, K13-6, K12-2, and K14-5 in fig. S4) and show some level of perturbation as indicated by the large mean square weighted deviation (MSWD) values, suggesting that their K-Ar systematics have been partially affected by subsequent impact events.

### Four- to 3-Ga dating results

Four of 28 samples fall into the 4- to 3-Ga age range, of which only two (K7-11 and K11-7) yield reliable ages of 3935 ± 152 and 3749 ± 31 Ma, respectively (Fig. 1 and fig. S5). Although the data points are concordant within a 2σ confidence interval, the large uncertainty of 152 Ma (sample K7-11) precludes their interpretation as a single population. Both K5-2 and K5-1 released insufficient Ar for extensive analysis and were analyzed for only three steps. Among these, only two consecutive steps with consistent results within error exist in K5-2, making it impossible to define a valid plateau age. Accordingly, these samples are not considered in the discussion.

### <3-Ga dating results

Fourteen of 28 samples are within the <3-Ga age range, ranging from 2936 ± 23 to 1130 ± 14 Ma (Fig. 1 and fig. S6). Although five of these ages broadly overlap with the ∼2.8-Ga formation age of the local CE-6 low-Ti mare basalt (*56*–*58*), their textures (K18-4, K14-6, K19-2, K14-7, and K11-1 in fig. S1) are distinct from the porphyritic or subophitic textures exhibited by CE-6 basalts (*43*, *56*–*58*), indicating that they are not pristine basalts. Their elevated Ir contents (ranging from 27.8 to 438 ppb, except K13-17, which has Ir lower than the detection limit; table S1) corroborate the impact melt origin (*51*). K18-4 and K14-7 compositionally resemble basaltic impact melts (fig. S2). K13-17 and K17-17 are plotted within the feldspathic highland terrane (FHT) region (fig. S2). Other samples exhibit characteristics consistent with nonmare impact melts (Fig. 2).

## DISCUSSION

Our results reveal a series of impact ages from 4330 ± 65 to 1130 ± 14 Ma (Fig. 1). Among these, three melt clasts are dated to 4153 ± 14 Ma. Notably, only two ages are identified in the 4.0 to 3.7 Ga, and notably no results are recorded between 3.7 and 3.0 Ga. Beyond this gap, a continuous distribution of impact ages resumes, extending through the Eratosthenian and Copernican periods to ∼1.13 Ga.

### The pre–4-Ga impact melt rocks

The major and trace element characteristics indicate that pre–4-Ga samples are both basaltic impact melt and nonmare impact melt, with three samples plotting within the FHT region (fig. S2). Their rare-earth element (REE) patterns exhibit both pronounced positive and negative Eu anomalies, respectively, resembling ferroan anorthite (FAN) and KREEP (potassium, REEs, and phosphorus) lithologies (fig. S3). The FANs are considered to be primary lunar flotation-cumulate crust (*59*, *60*) that crystallized in the late stages of magma ocean solidification, formed before the SPA impact event. Although there is still controversy over the scenario and timing of the formation of the Moon’s crust, it is certainly older than 4.16 Ga. It is therefore reasonable to infer that these pre–4-Ga samples formed before ∼4.16 Ga but had their K-Ar isotopic system subsequently reset by later impact event(s).

The CE-6 landed in the southern margin of the Apollo basin within the northeast region of the SPA basin (*61*). More than 72% of the impact melt rocks in the CE-6 returned samples are locally derived (*47*). These impact melt rocks could be either related to the formation of the SPA basin or to the Apollo basin. Given the relatively low closure temperature (<400°C) of the ^40^Ar/^39^Ar system (*42*, *62*, *63*), early-formed samples are prone to partial or complete resetting of their K-Ar isotopic system by subsequent impact events during the long history of lunar surface bombardment and the extent of resetting depending on the heating temperature and duration. However, plateau ages obtained on pre–4-Ga samples show that four samples were completely unaffected by subsequent impacts. How can we explain that the heat sensitive ^40^Ar/^39^Ar isotopic clock remained undisturbed in these four melt rocks during subsequent impact events? One possible explanation is that large impact event may have produced enough melt volume. This early-formed melt was then continuously exhumed and brought to the surface through repeated excavations by subsequent impacts. Simulation results indicate that some of these early-formed impact melt rocks were not fully disrupted by subsequent reexcavation events, thereby remaining undisturbed at the lunar surface (*32*). Alternatively, because no large impact basin occurred around the CE-6 landing site (*64*) after this period, the early impacts can be preserved in contrast to the nearside where the Imbrium impact event may have masked impact melt rocks from other events (*27*). Smaller impact events occurred over the history of this region, which erased only the record of local target rocks proximal to the impact while simultaneously exhuming deeper, unaffected impact melt rocks. Therefore, both ∼4.33- and ∼4.16-Ga ages likely represent the formation timing of major basins or at least large impacts, both of which are discussed in detail below.

Pb-Pb dating of two impact melt rock fragments has suggested that Apollo basin was formed at 4165 ± 14 Ma (*14*). Considering the high closure temperature of the Pb-Pb system in Zr-bearing minerals [>600°C (*65*)], it is not evident if the Pb-Pb age necessarily records an impact event, represents a partially reset age, or reflects a crystallization age. The ^40^Ar/^39^Ar system, on the other hand, is very sensitive to resetting by impact events of even moderate size particularly for melt rocks (*62*). Our ^40^Ar/^39^Ar ages of 4174 ± 40, 4156 ± 17, and 4128 ± 31 Ma are interpreted to reflect a single impact event at 4153 ± 14 Ma. These three ages are indistinguishable with the Pb-Pb age [4165 ± 14 Ma (*14*)], and the four ages yield a weighted mean age of 4159 ± 10 Ma (*n* = 4; *P* = 0.14). These four ages are obtained using two different isotopic clocks on five distinct impact melt fragments, providing confidence that the formation age of the Apollo basin is ∼4.16 Ga.

Nevertheless, one melt rock (sample K11-3) yields an even older age of 4330 ± 65 Ma (Fig. 1 and fig. S4). The chondrite-normalized REE pattern of this sample is characterized by strong LREE enrichment, a pronounced negative Eu anomaly, and fractionated HREEs. This REE pattern is inconsistent with FAN or low-Ti mare basalt sources but is consistent with melts derived from Mg-suite/KREEP lithologies (fig. S3). Two possible explanations can be conceived: (i) This age dates the formation of the SPA basin itself; (ii) it points to the formation of an older impact or basin whose surface expression has been erased by subsequent impact processes.

Crater-counting studies suggest that SPA basin is older than ∼4.2 Ga (*66*). Analysis of NWA2995 meteorite suggested that the formation age of SPA basin is ∼4.33 Ga (*36*), which is virtually identical to our ^40^Ar/^39^Ar age. However, five distinct noritic clasts recovered by the CE-6 mission (*35*) suggested that the SPA was formed at ∼4.25 Ga. Although the age obtained from sample K12-3 (4260 ± 127 Ma) is consistent within error with the Pb-Pb age (4247 ± 5 Ma) from CE-6 norite clasts (*35*), the large analytical uncertainty precludes a robust assessment of whether these two ages represent the same event. Consequently, no unequivocal age of ∼4.25 Ga was obtained in our dataset. The absence of ∼4.25-Ga age in our dataset is intriguing and can be accounted for by two possible scenarios. (i) The SPA basin was formed at ∼4.25 (*35*), but the K-Ar signal of that event has been totally reset by the Apollo basin impact at 4.16 Ga. However, this interpretation is apparently at odds with the preservation of older thermal events at ∼4.33Ga. (ii) The SPA basin was formed at ∼4.33 Ga (*36*), and the ∼4.25 Ga may represent an intermediate event between the Apollo and SPA impacts. In particular, the noritic impact melts, which contain ∼4.25-Ga Zr-bearing phases, are not local to the CE-6 landing site but mostly likely represent ejecta from impact outside of the SPA basin. This is supported by the fact that the ∼4.25-Ga noritic impact rocks show no KREEP signal (*35*), at odds with the moderate levels of thorium (Th) contents in the SPA region (*67*). In contrast, both NWA2995 meteorite (*68*) and ∼4.16-Ga impact melt clasts (*14*) carry a KREEP signature, consistent with a derivation from the SPA and/or Apollo basin. Regardless of which scenario proves to be the case, this impact melt clast (sample K11-3) with an age of ∼4.33 Ga was generated in crustal terranes containing Mg-suite intrusive and KREEP components, later ballistically ejected into the Apollo crater. Its survival and its age older than the Apollo basin make it the first direct farside evidence of large-scale impacts into Mg-suite and KREEP crustal domains, extending the pre-Nectarian impact chronology.

### Reevaluating the LHB hypothesis

**Fig. 3.**
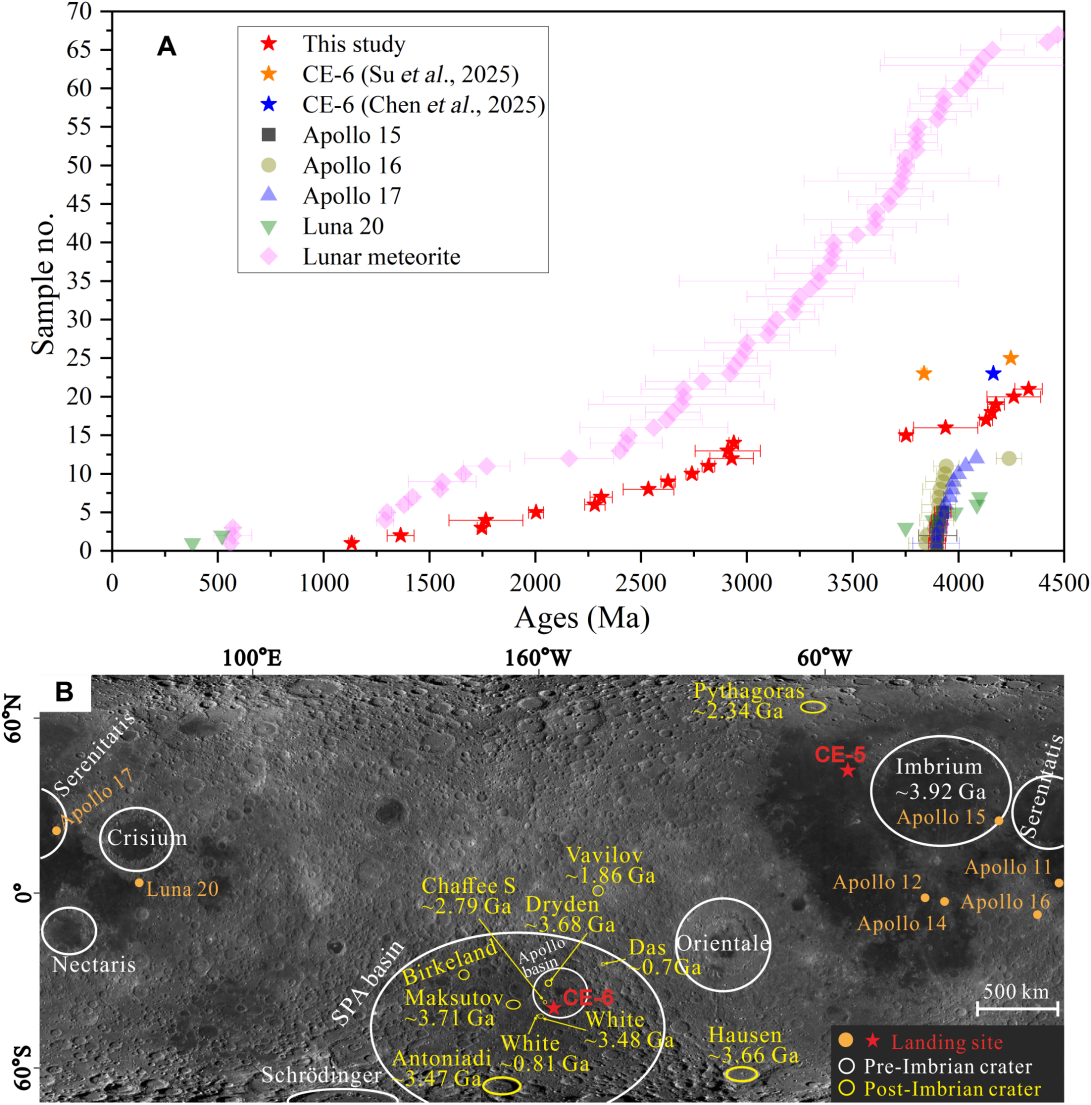
Statistic of the ^40^Ar/^39^Ar ages of impact melt rocks from the Moon. (**A**) The published ^40^Ar/^39^Ar ages of Apollo, Luna, and meteorite are compiled from Nemchin *et al.* (*27*) and Michael *et al.* (*32*), respectively. The red star are the results in this study. The orange and blue stars denote the reported Pb-Pb ages for CE-6 impact melt rocks from Su *et al.* (*35*) and Chen *et al.* (*14*). (**B**) The geological context around the CE-6 landing site. The background is downloaded from Lunar QuickMap (https://quickmap.lroc.im-ldi.com). We acknowledge the use of imagery from this platform, a collaboration between NASA, Arizona State University, and Applied Coherent Technology Corp. Ages of relative crater are from Xu *et al.* (*64*).

Several hypotheses have been proposed for the early impact history of the Moon (e.g., cataclysmic LHB, sawtooth-like, and accretion tail) (*10*, *16*, *23*, *24*, *32*). The formulation of these different models and the ongoing debate largely stems from the fact that lunar impact samples collected from multiple Apollo landing sites (Fig. 3) have been severely biased by the ejecta from Imbrium basin formation (*27*, *29*, *31*), resulting in a limited number of reliable ages > 4.0 Ga, coupled with the unexpectedly consistent isotopic disturbances recorded at 4.0 to 3.9 Ga (Fig. 3). ^40^Ar/^39^Ar dating of impact melt rocks directly collected by the CE-6 mission from lunar far side shows no clear concentration of ages around ∼3.9 Ga, with only two such ages (3935 ± 152 and 3749 ± 31 Ma; Fig. 1 and fig. S5) among 21 meaningful ^40^Ar/^39^Ar plateau ages. Therefore, based on (i) older radioisotopic ages of the major farside basins, ∼4.16 Ga for Apollo, which firmly extend the basin-forming epoch back well before 3.9 Ga, (ii) the existence of even older preserved impact melt rocks with an age of ∼4.33 Ga, and (iii) the near-absence of robustly dated melt rocks with ages ∼3.9 Ga from our ^40^Ar/^39^Ar dating results, we further demonstrated that the farside lunar bombardment record is inconsistent with a narrow, cataclysmic LHB. Instead, these data support a scenario of a smooth, long-term decline in impact flux from the earliest basin-forming events (>4.3 Ga) through to the Nectarian and early Imbrian, with no requirement for a sharp spike in basin-forming activity at ∼3.9 Ga (*69*).

### The lunar farside bombardment history

The overwhelming influence of Imbrium impact event on the lunar nearside may have masked the true lunar bombardment history (*27*–*29*, *31*). The first lunar farside samples returned by the CE-6 mission offer the potential to reconstruct the true history of lunar bombardment because the lunar farside escaped the Imbrium event. Our data show that ∼3.9-Ga ages are almost absent from the studied impact melt rocks. This contrasts with the significant bias toward Imbrium ejecta observed in Apollo collection (*27*, *70*) and suggests that the CE-6 samples of impact melt rocks are not dominated by Imbrium-related materials. Therefore, these samples offer an opportunity to discuss in more details the pre–3.9-Ga bombardment history of the moon.

Beyond their significance for the impact history before 3.9 Ga, the CE-6 impact melt rocks are also important for understanding the <4-Ga impact record. Figure 3A compares ^40^Ar/^39^Ar ages of impact melt rocks from Apollo samples, meteorites, and CE-6 samples. Two interesting features are evident from our data. The first one is the absence of any data between 3.7 and 3.0 Ga. Although the number of samples is still small, this time gap is hard to explain by only invoking a sample bias, especially considering that the record from 2.9 to 1.1 Ga is continuous and may suggest that the record has been erased by ≤2.9-Ga impact events. This is especially plausible since the number of basin-forming events after 3.7 Ga was limited and especially on the farside due to its thicker crust (*71*, *72*). Looking more closely at the source of the nonmare materials in CE-6, the exotic components were mainly transferred from White, Vavilov, Pythagoras, and Chaffee S craters (*47*), with the model ages of impact craters being 0.805 ± 0.05, 1.86 ± 0.12, 2.34 ± 0.17, and 2.79 ± 0.28 Ga, respectively (Fig. 3B) (*64*). The samples contain very little material from the 3.7- and 3.0-Ga impact events (e.g., Antoniadi, White, Hausen, and Dryden craters) (*64*). A lower amount of material would imply a lower probability of preservation from subsequent impacts (*32*). This is consistent with the absence of ages within this interval in our dataset.

The other feature is that after ∼2.9 Ga, the record is somewhat continuous down to 1.1 Ga. This suggests that impact occurred on a regular basis and indicates limited sample bias for this time period. In any case, the farside record diverges markedly from the nearside one, where ages around ∼3.9 Ga dominate because of the Imbrium overprint. The nonmare ejecta in the CE-6 soils includes materials from many impact events that most likely occurred on the far side. This is supported by very little ∼3.9-Ga materials identified in our sample. Our data further show that in a clear impact record (i.e., that is not obscured by Imbrium material), even very small-scale impact events are likely to have their products preserved and identified in the lunar regolith. This highlights the value of farside sampling for reconstructing a more representative lunar bombardment chronology.

## Supplementary Materials

### The PDF file includes:

Supplementary Text Figs. S1 to S8 Legends for tables S1 and S2 References
