# Supplementary material for: The bombardment history on the lunar farside revealed by 40Ar/39Ar geochronology of Chang’e-6 impact melt rocks
Source: Sci Adv. 2026 Jul 23;12(30):eaee8718. doi: 10.1126/sciadv.aee8718 (PMC13394471; doi:10.1126/sciadv.aee8718)
Supplement: Supplementary file 1 — Supplementary Text Figs. S1 to S8 Legends for tables S1 and S2 References [file sciadv.aee8718_sm.pdf]

Science Advances
AAAS

Supplementary Materials for

The bombardment history on the lunar farside revealed by $^{40}Ar/^{39}Ar$ geochronology of Chang'e-6 impact melt rocks

Wan-Feng Zhang *et al.*

Corresponding author: Wan-Feng Zhang, wfzhang@gig.ac.cn; Yi-Gang Xu, yigangxu@gig.ac.cn

Sci. Adv. **12**, eaee8718 (2026)
DOI: 10.1126/sciadv.aee8718

The PDF file includes:

Supplementary Text
Figs. S1 to S8
Legends for tables S1 and S2
References

Other Supplementary Material for this manuscript includes the following:

Tables S1 and S2

Supplementary Text

Electron microprobe analysis of minerals method

The studied sample clasts were embedded in adhesive mounts (Crystalbond ™ 509, which is soluble in acetone) and subsequently polished using a grinder with fine diamond pastes (1 μm). The samples mounts were coated with a carbon layer prior to scanning electron microscope (SEM) analyses. Backscattered electron (BSE) images of the clasts were obtained using a Carl Zeiss SUPRA55SAPPHIRE Field Emission SEM at the State Key Laboratory of Deep Earth Processes and Resources Guangzhou Institute of Geochemistry, Chinese Academy of Sciences (GIGCAS). Typical impact melt rocks in Apollo and Luna samples were crystalline, fine-grained, microporphyritic, and plagioclase rich (*30, 33, 48*). Thus, clasts exhibiting BSE image characteristic similar to those of impact melt clasts identified in previous studies were selected for this study (Fig. S1). Prior to extraction, the carbon coating on the sample surface was completely removed by polishing. The adhesive surrounding the selected sample clast was locally melted using a heating needle at 180-200 °C, allowing the clast to be extracted from the sample mounts and transferred into numbered centrifuge tubes. Individual sample clasts were cleaned using acetone for 10 min in an ultrasonic bath (three times) to remove any potential residual mounting material and dried in an oven (50 °C, >4 hours).

$^{40}Ar/^{39}Ar$ dating method

Each clast was individually packed in aluminum foil (Fig. S7A) and successively loaded into the quartz tubes (Fig. S7B). Prior to irradiation, the quartz tubes were vacuum-sealed. The ZMT04 muscovite (J value monitor, 1772.2 ± 2.7 Ma, 2σ (*73*)), HB3gr hornblende (monitor sample, 1081.0 ± 2.4 Ma, 2σ (*74*)) and WA1ms muscovite (monitor sample, 2613.0 ± 4.8 Ma, 2σ (*75*)) were loaded into the quartz tube in a specific order. The quartz tube was vacuum sealed and then placed in the Cd-shielded aluminum tube holder. The samples were irradiated in High Flux Engineering Test Reactor (Sichuan, China) for 14.5 hours, based on calculations from our previous study to ensure sufficient ^{39}Ar production for Precambrian samples (*73, 76*).

Following irradiation, all standard and sample clasts were individually transferred to a copper disc (Extended Data Fig. S7C), with documentation to ensure that each analytical result could be correctly linked to its corresponding clast. Both samples and standards were analyzed at GIGCAS, using an Argus VI mass spectrometer (Thermo Fisher Scientic Inc.) in multi-collection mode using

two Faraday cups each equipped with a 10^{12} ohm resistor amplifier for masses 40 and 36, and three Faraday cups equipped with 10^{13} ohm resistor amplifier for masses 39, 38 and 37. The sample was subjected to stepwise heating by continuously firing a 50 W custom-made CO_2 laser (IR, 10.6 μm) for 100 seconds. The released gases were initially cleaned using a cryotrap (-70 °C) to absorb the moisture, followed by further purification with two custom-made Zr/Al getters at 400 °C and one in room temperature. During the stepwise heating process, blanks were analyzed every two sample steps and typical ^{40}Ar, ^{39}Ar and ^{36}Ar blank values were ca. 26.5, 0.175, and 0.472 fA, respectively. The mass discrimination factor was monitored regularly through the analysis using an automated air pipette and provided values of 0.992101 to 0.996586 per Dalton relative to an atmospheric argon ratio of 298.56 ± 0.31 *(77)*. The total decay constant for ^{40}K of $(5.531 \pm 0.013) \times 10^{-10}$ a^{-1} is recommended by Renne et al. *(74)*. The interference correction values of the reactor are $(^{36}Ar/^{37}Ar)_{Ca} = (2.468 \pm 0.039) \times 10^{-4}$, $(^{39}Ar/^{37}Ar)_{Ca} = (8.174 \pm 0.082) \times 10^{-4}$, and $(^{40}Ar/^{39}Ar)_K = (3.02 \pm 0.73) \times 10^{-3}$. In addition, $(^{38}Ar/^{36}Ar)_{cos}$ and $(^{38}Ar/^{36}Ar)_{tr}$ were adopted as 1.54 *(78)* and 0.188 *(79)*, respectively. Trapped intercept $^{40}Ar/^{36}Ar$ ratio ($(^{40}Ar/^{36}Ar)_{tr}$) were measured using the inverse isochron method according to previous study *(54, 55, 62, 80-82)*. During data processing, we first perform a least-squares linear regression using as many points as possible to obtain the initial $^{40}Ar/^{36}Ar$ ratio, while excluding points that deviate significantly from the fitted curve. This initial $^{40}Ar/^{36}Ar$ ratio is then used to calculate the age plateau. We then examine whether the steps are continuous and consistent within error. If any step deviates significantly, it is excluded, and the isochron is refitted to recalculate the initial $^{40}Ar/^{36}Ar$ ratio. This new ratio is applied to reprocess the plateau data, and the iteration continues until neither the plateau nor the isochron intercept changes further. The J-values of the samples were obtained from the J-value curves (J-value = -0.000017218269×sample position (mm) + 0.047746121227) derived from the ZMT04 muscovite samples (1772.2 ± 2.7 Ma, *(73)*). The vertical distance from the bottom of the irradiation tube to the sample position during irradiation is listed for all samples, blind samples, and standards in Table S2. Data processing was performed using the software ArArCALC (Version 2.5.2, *(83)*). To avoid the misinterpretation that may arise from mixing robust ages with unreliable data, strict data quality criteria were used according to previous study *(27, 53)*. Ages derived from at least three consecutive steps comprising 50-70% (plateau) or >70% (robust plateau) of the released ^{39}Ar and that yield a probability of $P \geq 0.05$ are considered meaningful. Results that do not satisfy these

data quality criteria are regarded as unreliable. All data herein are reported with 2σ uncertainties including the J uncertainty.

Three grains of HB3gr hornblende and WA1ms muscovite were analyzed as monitor samples under the same conditions and procedures applied to the CE-6 samples. The $^{40}Ar/^{39}Ar$ plateau age obtained for K13-2 (HB3gr) and K13-1 (WA1ms) are 1075 ± 8 Ma and 2609 ± 6 Ma (Fig. S8), respectively. These results are consistent with recommended plateau ages of 1081.0 ± 2.4 Ma (*74*) and 2613.0 ± 4.8 Ma (*75*), confirming that the entire analytical process is reliable and free from artificial bias.

Major and trace elements analytical method

After completion of single clast $^{40}Ar/^{39}Ar$ dating analysis, each clast was fused into a glass bead. Once the entire batch of samples had been analyzed, the copper holder was removed from sample chamber. Sample clasts were retrieved from the holder, mounted on adhesive tape successively and embedded in epoxy resin and polished for major and trace element analysis by LA-ICPMS. This analytical procedure was validated using five reference materials (BCR-2G, BHVO-2G, TB-1, NIST SRM 610 and 612) that were processed following the same laser heating protocol used for $^{40}Ar/^{39}Ar$ dating, to evaluate elemental changes following CO_2 laser heating. For all elements except the Na, K, Zn, Pb, and Cu, measurements were within 10% of certified values, ensuring accurate quantification. Details of the experimental procedure and the corresponding evaluation are given in Zhang et al. (*49*).

All major and trace elements were measured with an ELEMENT XR (Thermo Fisher Scientific) ICP-SF-MS coupled with a 193-nm (ArF) Resonetics RESOlution M-50 laser ablation system in the GIGCAS. Laser condition was set as follows: 45 μm beam size, 6Hz repetition rate, ~4 J cm^{-2} energy density. A signal-smoothing device (the Squid, Laurin Technic) was used to smooth the sample signal during measurements with a repetition rate <10 Hz. Each spot analysis consists of 20 s gas blank collection with the laser off, and 30 s sample signal detection with the laser on. Signals of the following masses were detected: ^{29}Si, ^{31}P, ^{45}Sc, ^{49}Ti, ^{89}Y, ^{93}Nb, ^{139}La, ^{140}Ce, ^{141}Pr, ^{145}Nb, ^{149}Sm, ^{151}Eu, ^{155}Gd, ^{159}Tb, ^{163}Dy, ^{165}Ho, ^{167}Er, ^{169}Tm, ^{172}Yb, ^{175}Lu, ^{178}Hf, ^{232}Th, and ^{238}U. Si was selected as the internal standard element. NIST SRM 610 glass standard was selected as the calibration standard. The oxide molecular yield, indicated by the $^{238}U^{16}O/^{238}U$ ratio, was

less than 0.3%. Repeated analyses of BCR-2G and NIST SRM 612 reference glasses indicated that the accuracy and precision (2rsd) was better than 10%. The detailed experiment procedure and data reduction strategy are described in Zhang et al. (*84*).

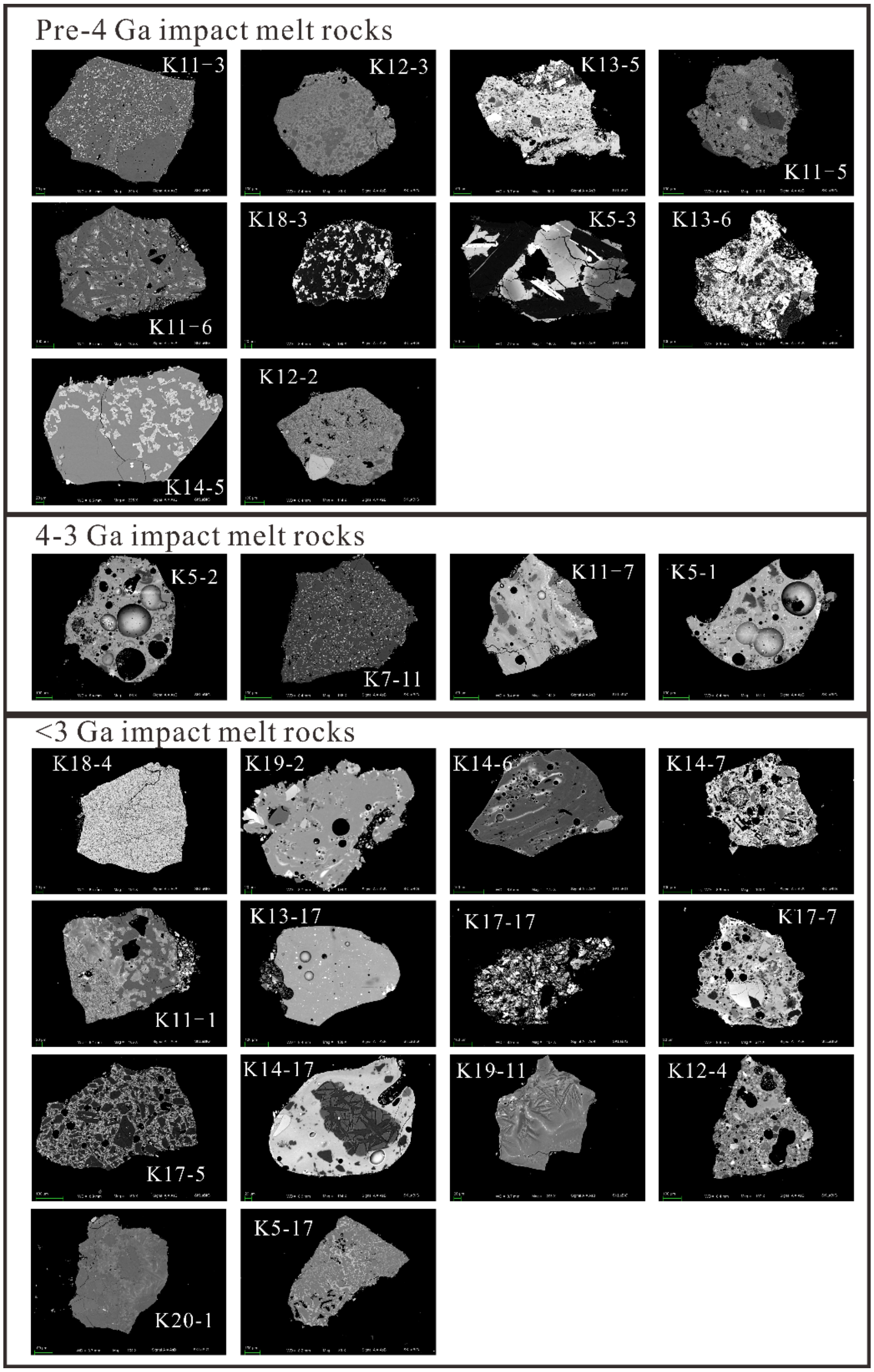

Fig. S1. BSE images of impact melt rock in all studied samples, arranged in order of $^{40}Ar/^{39}Ar$ age.

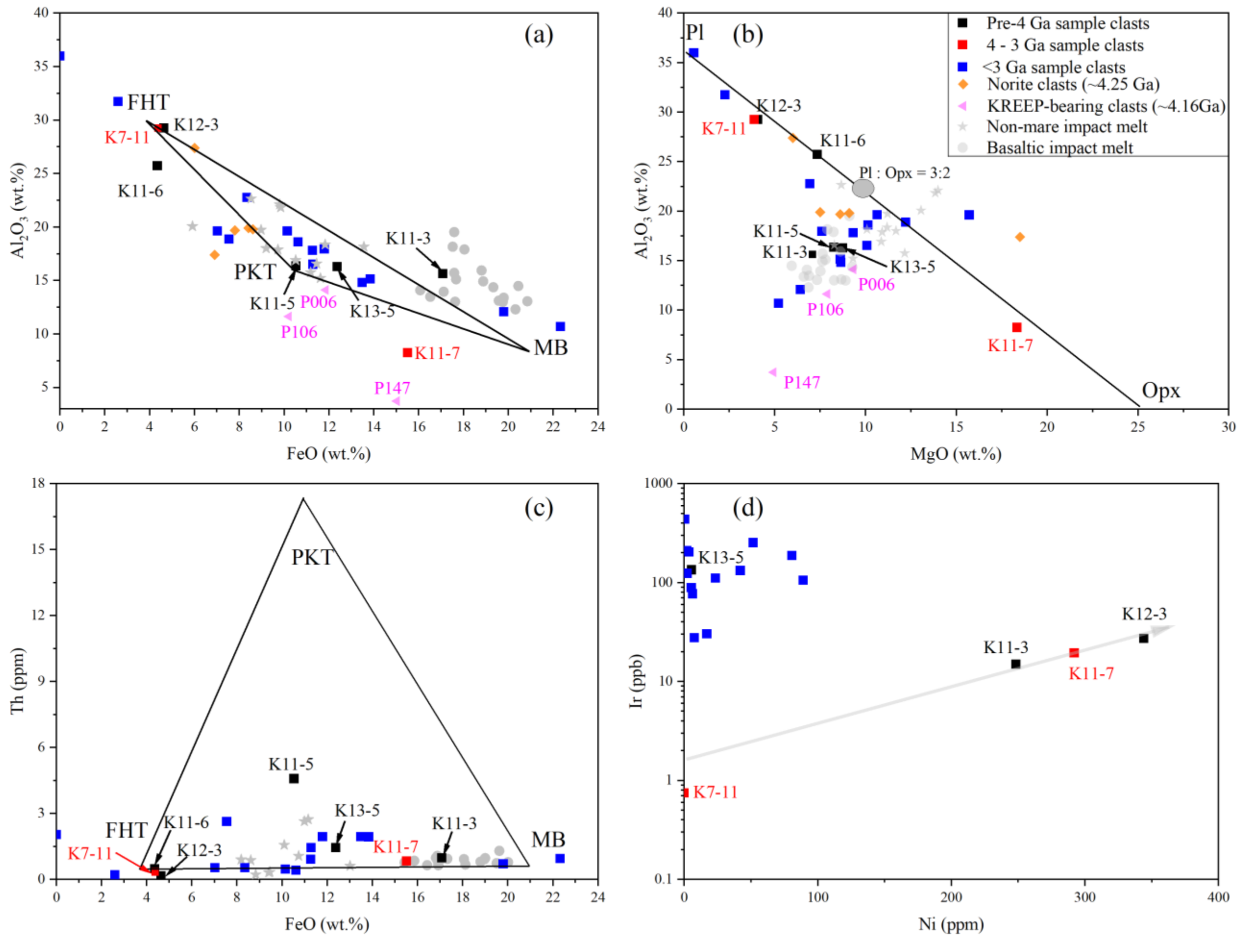

Fig. S2. Bulk major elements of CE-6 impactite; MB: Mare basalts; FHT: feldspathic highlands terrane; PKT: Procellarum KREEP terrane; Opx: orthopyroxene; Pl: plagioclase; Norite clasts(~4.25 Ga, Su et al. (35)); KREEP-bearing clasts(~4.16 Ga, Chen et al. (14)).

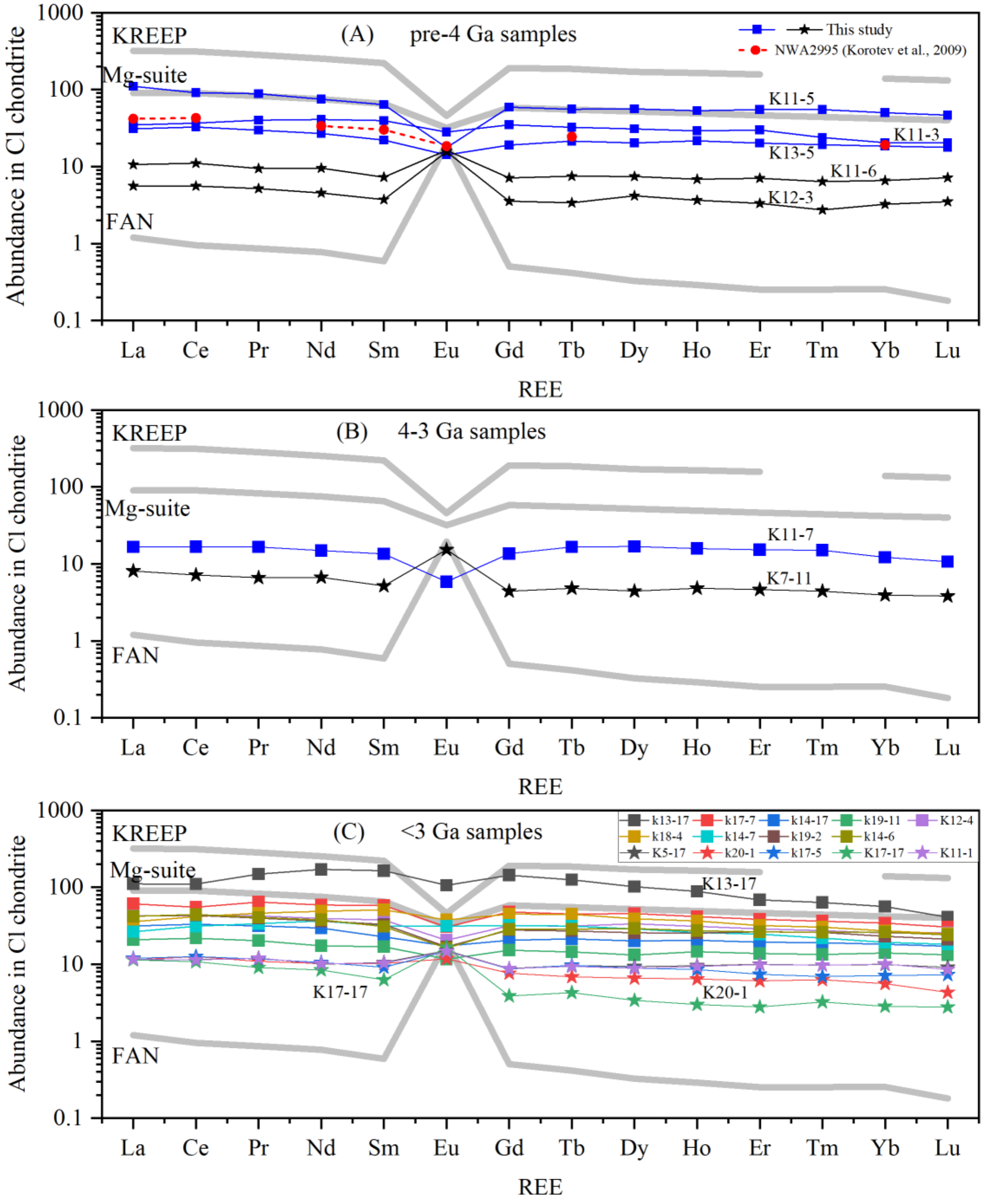

Fig. S3. Rare earth element (REE) of CE-6 impact melt rocks. FAN: ferroan anorthosite; Mg-suit: magnesian-suit; KREEP: potassium, rare earth elements, and phosphorus. The black and blue lines represent results with and without a higher Eu anomaly, respectively. Data source: NWA2995 (68); KREEP basalt (Apollo 15, 15386_54, ***(85)***); FAN data (60025, ***(86, 87)***), and Mg-suite (15445, ***(88)***).

Pre-4 Ga impact melt rocks results (4.33 - 4.13 Ga)

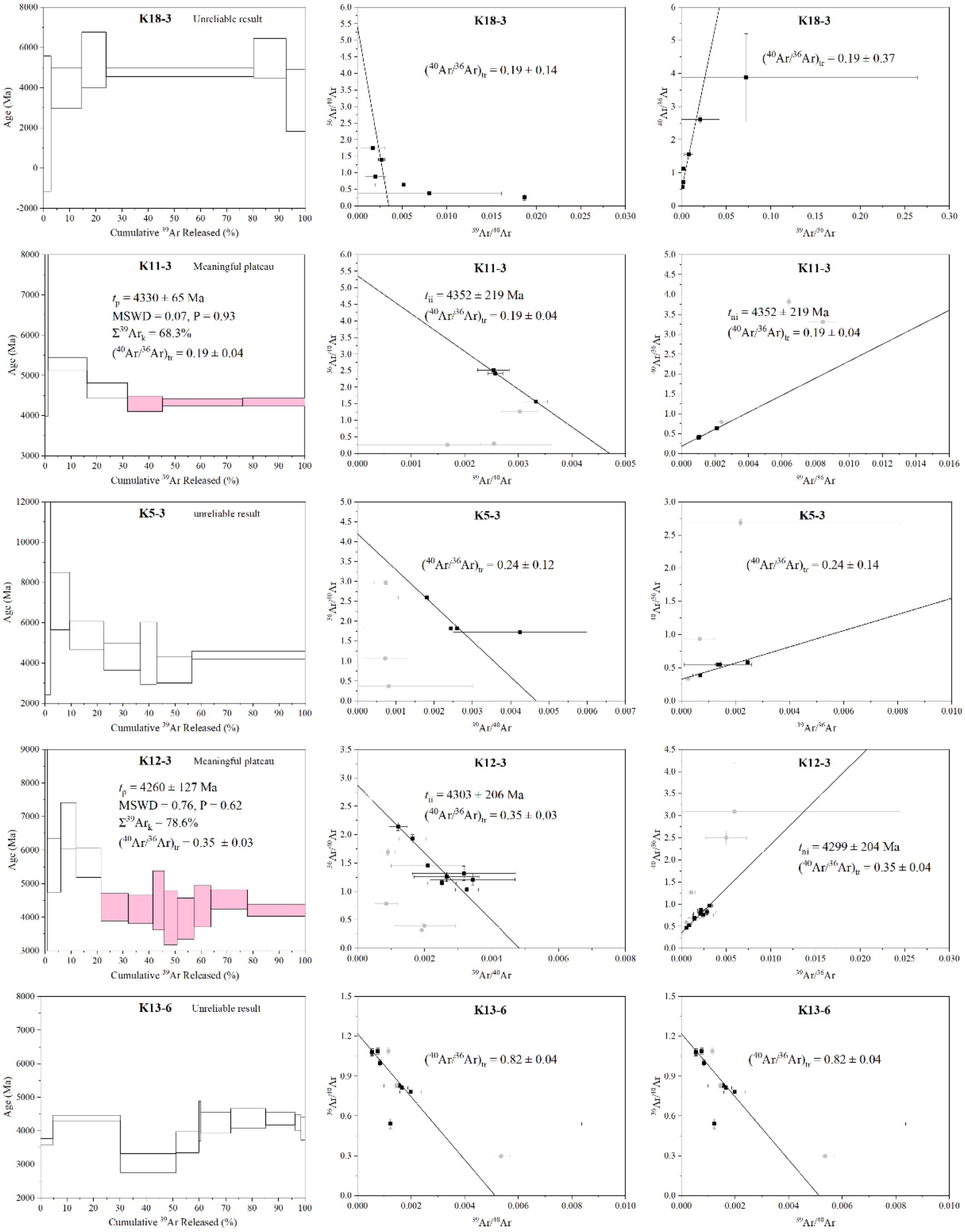

Fig. S4-1. $^{40}Ar/^{39}Ar$ age spectra of pre-4 Ga samples. Samples K18-3, K5-3, and K13-6 did not yield meaningful ages as they failed to meet the robust plateau (>70% ^{39}Ar released) or plateau (50-70% ^{39}Ar released) criteria. Their age results are therefore not shown, and they are displayed without color fill.

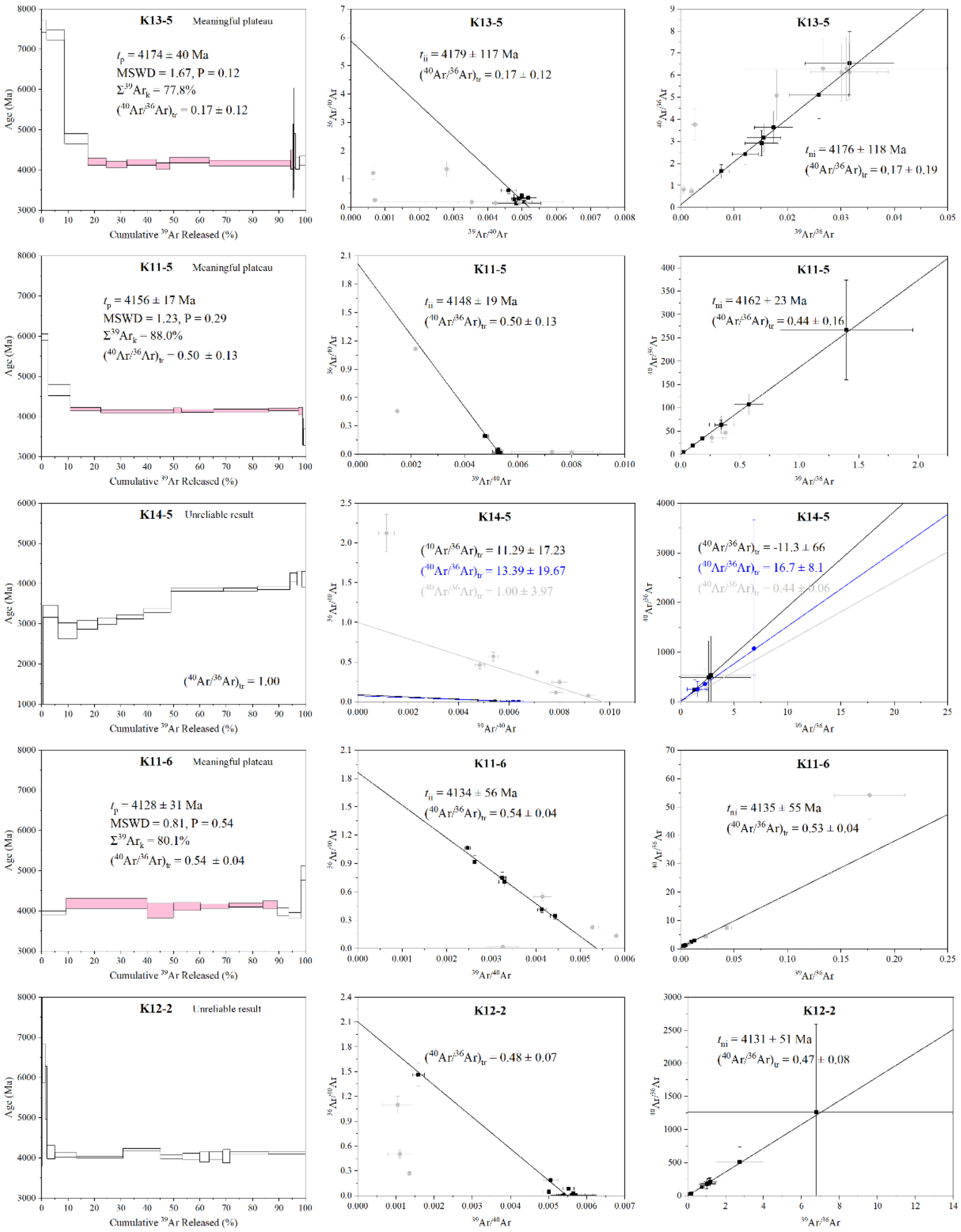

Fig. S4-2. $^{40}Ar/^{39}Ar$ age spectra of pre-4 Ga samples results. Samples K14-5 and K12-2 did not yield meaningful ages as they failed to meet the plateau criteria, and are therefore not shown.

4-3 Ga impact melt rocks results (3.94 - 3.75 Ga)

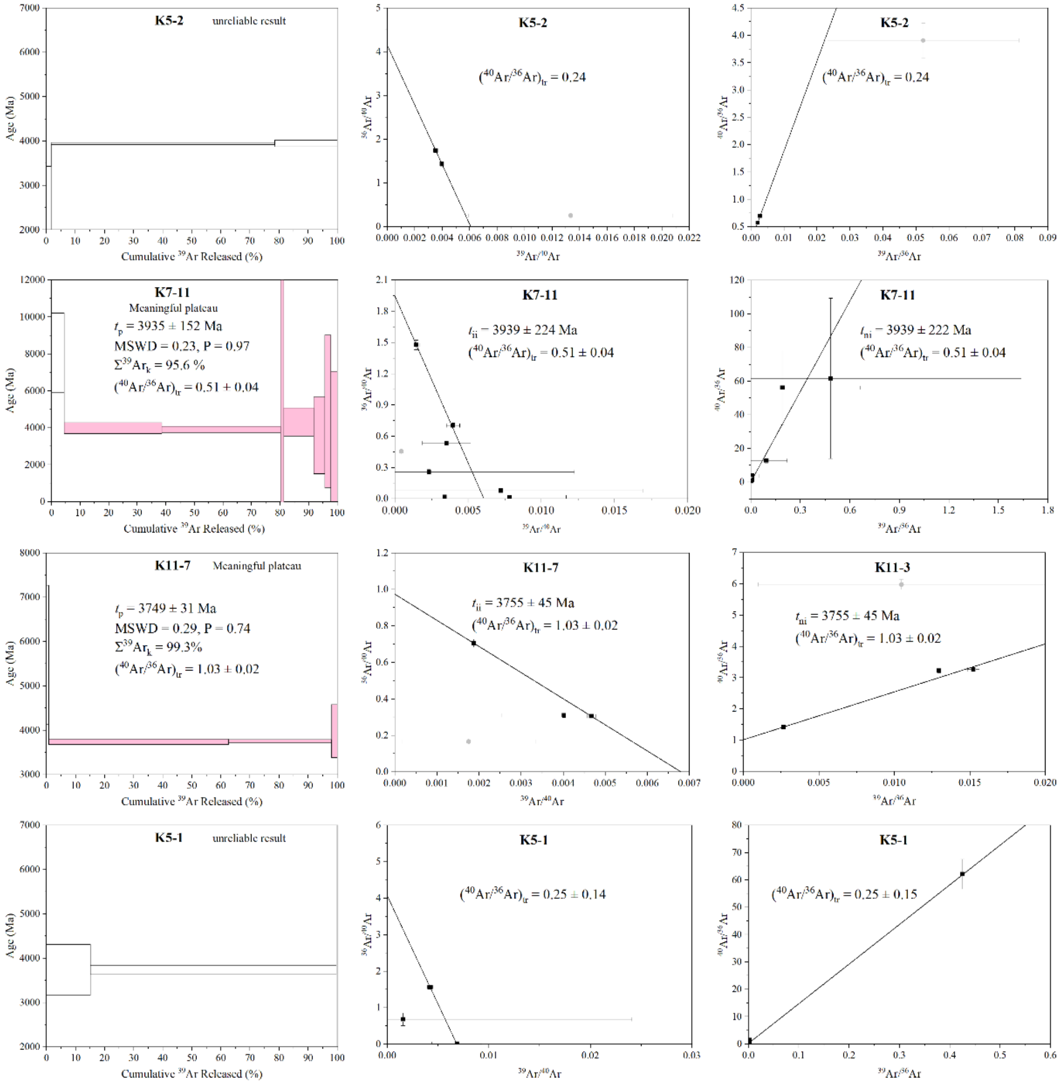

Fig. S5. $^{40}Ar/^{39}Ar$ age spectra of 4-3 Ga impact melt rocks sample results. Samples K5-2 and K5-1 did not yield meaningful ages as they failed to meet the plateau criteria, and are therefore not shown.

<3 Ga impact melt rocks results

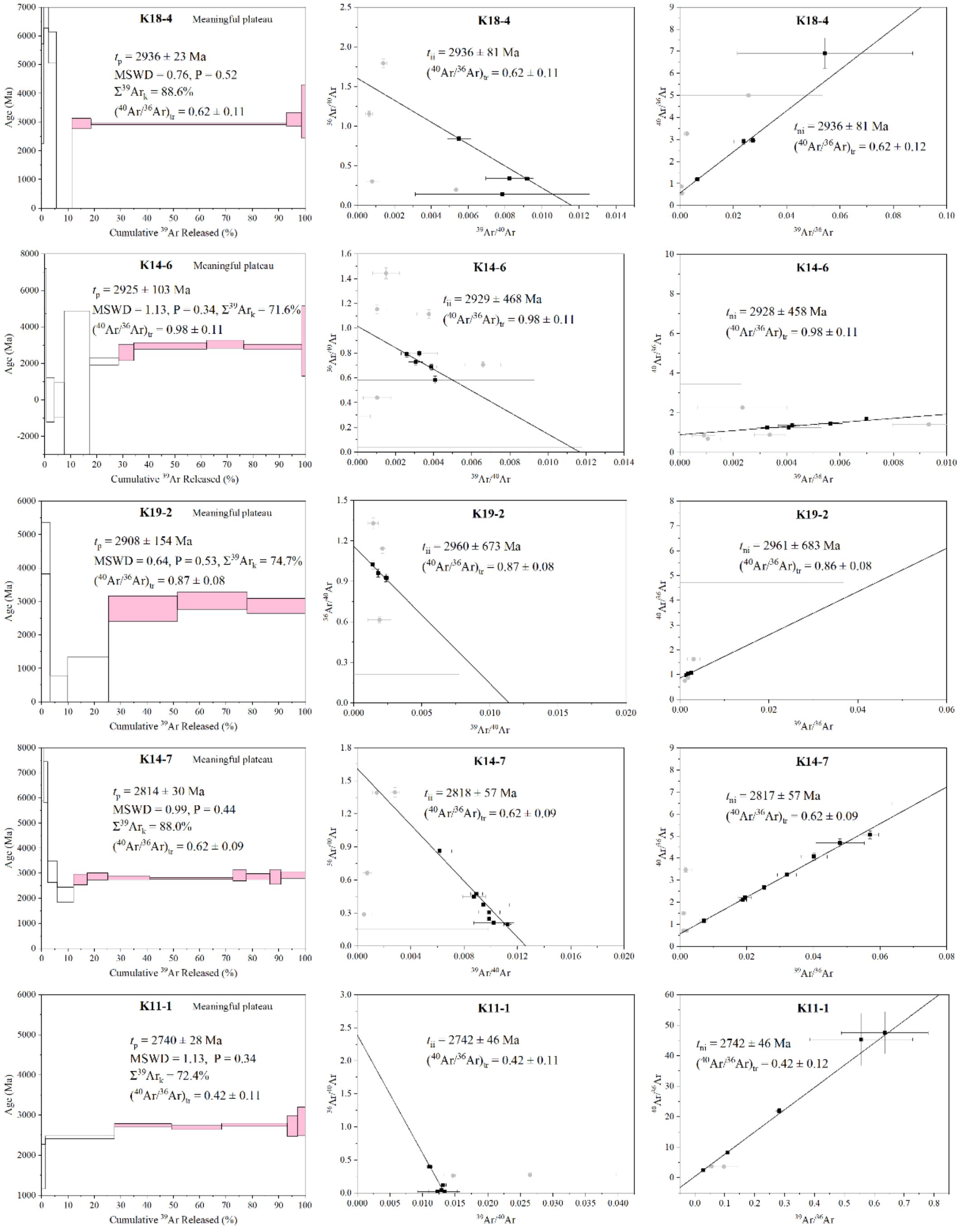

Fig. S6-1. $^{40}Ar/^{39}Ar$ age spectra of <3 Ga sample results.

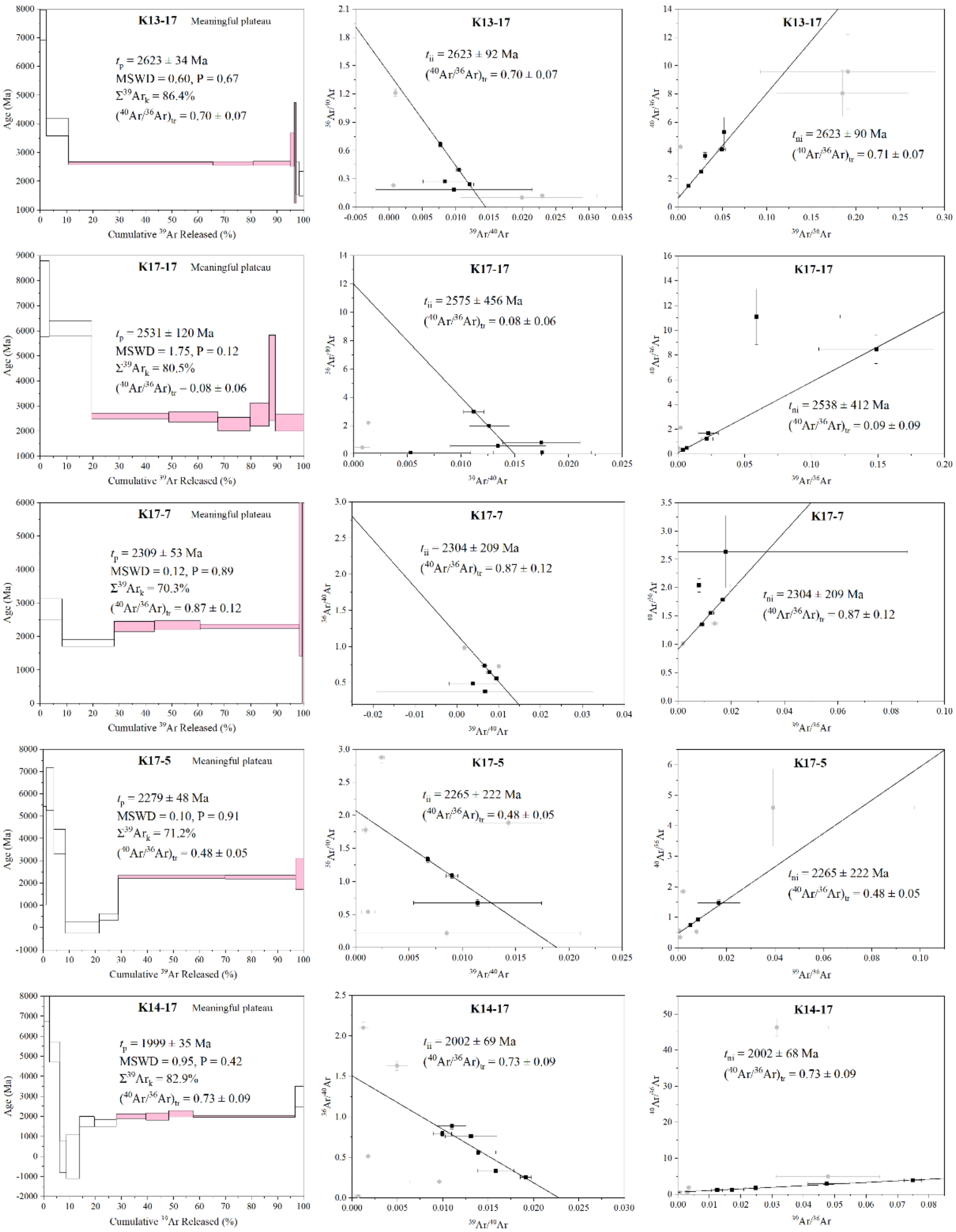

Fig. S6-2. $^{40}Ar/^{39}Ar$ age spectra of <3 Ga sample results.

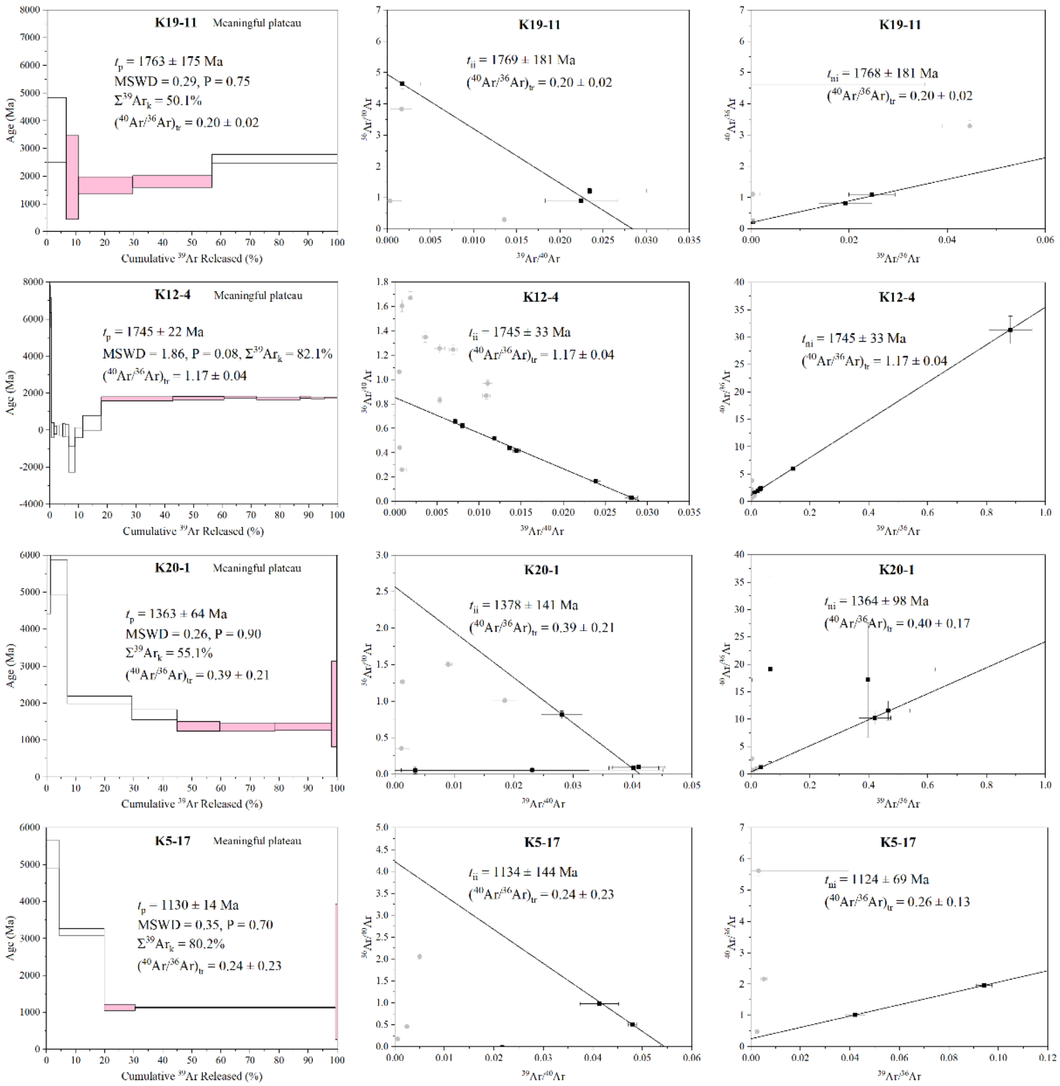

Fig. S6-3. $^{40}Ar/^{39}Ar$ age spectra of <3 Ga sample results.

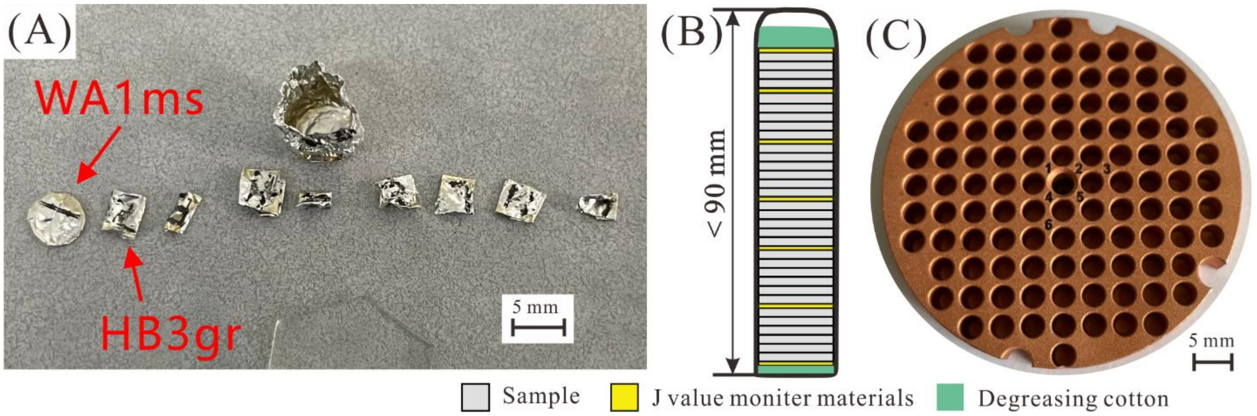

Fig. S7. Sample preparation process. (A): Single clast packaged; (B): Schematic diagram showing the sample loading pattern in the quartz tube; (C): Transferring irradiated clasts one by one onto a copper disk for argon analysis;

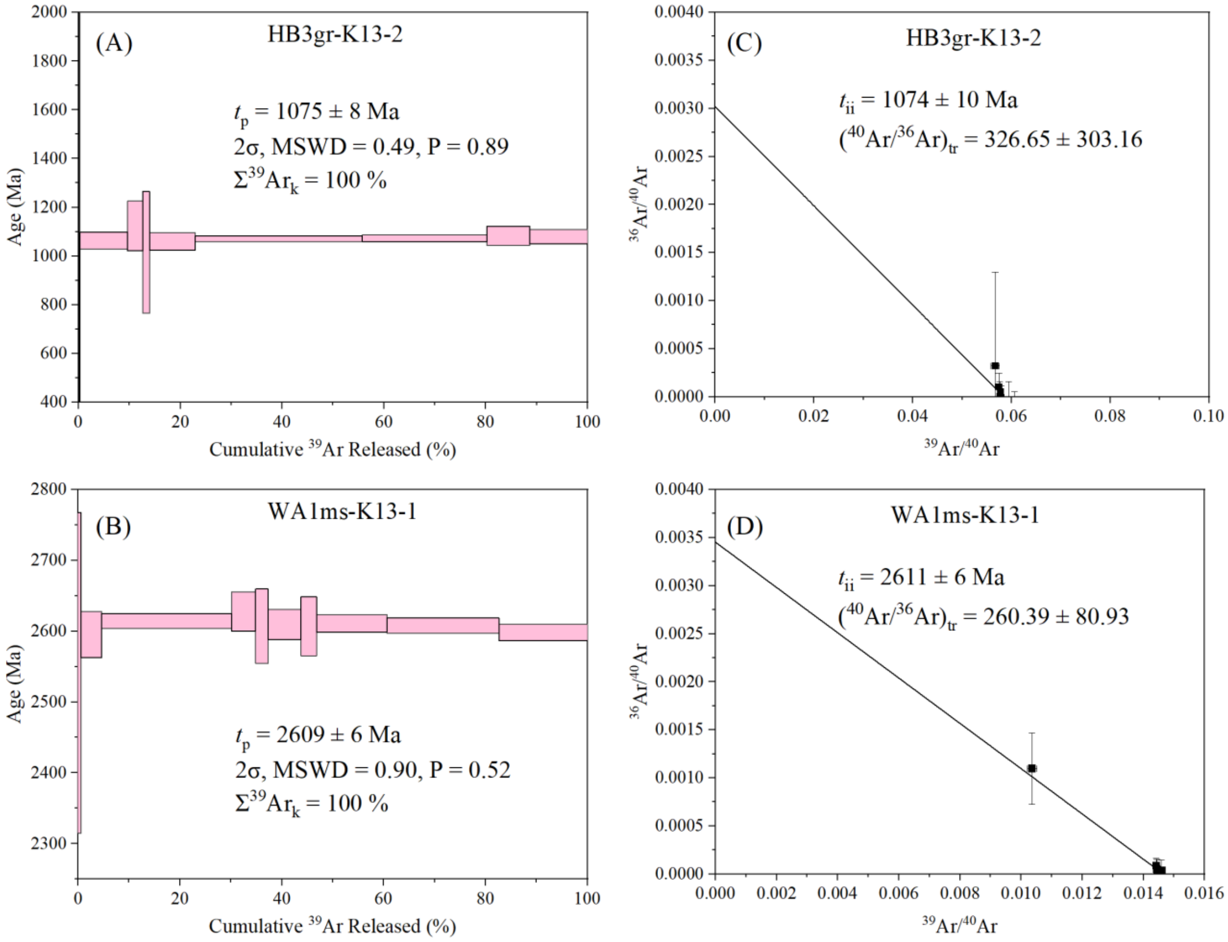

Fig. S8. $^{40}Ar/^{39}Ar$ plateau age (A & B) and inverse isochron (C & D) of two blind samples (HB3gr and WA1ms).

Caption of the supplementary Table

Table S1. (.xlsx file)

Table S1. Major and trace element composition of the impact melt rocks

Table S2. (.xlsx file)

Table S2 Complete $^{40}Ar/^{39}Ar$ raw data of the standard and CE-6 impact melt rocks

REFERENCES

1. G. Turner, Argon-40/ argon-39 dating of lunar rock samples. *Science* **167**, 466–468 (1970).

2. G. Turner, P. H. Cadogan, C. J. Yonge, Argon selenochronology. *Geochim. Cosmochim. Acta* **2**, 1889–1914 (1973).

3. E. K. Jessberger, J. C. Huneke, G. J. Wasserburg, Evidence for a - 4.5 aeon age of plagioclase clasts in a lunar highland breccia. *Nature* **248**, 199–202 (1974).

4. F. Tera, D. A. Papanastassiou, G. J. Wasserburg, Isotopic evidence for a terminal lunar cataclysm. *Earth Planet. Sci. Lett.* **22**, 1–21 (1974).

5. G. W. Wetherill, Late heavy bombardment of the moon and terrestrial planets. *LPI* **6**, 866–868 (1975).

6. W. F. Bottke, M. D. Norman, The late heavy bombardment. *Annu. Rev. Earth Planet. Sci.* **45**, 619–647 (2017).

7. G. Ryder, Mass flux in the ancient Earth-Moon system and benign implications for the origin of life on Earth. *J. Geophys. Res. Planets* **107**, 5022 (2002).

8. S. Charnoz, A. Morbidelli, L. Dones, J. Salmon, Did Saturn's rings form during the Late Heavy Bombardment? *Icarus* **199**, 413–428 (2009).

9. J. Fritz, B. Bitsch, E. Kuehrt, A. Morbidelli, C. Tornow, K. Wuennemann, V. A. Fernandes, J. L. Grenfell, H. Rauer, R. Wagner, S. C. Werner, Earth-like habitats in planetary systems. *Planet. Space Sci.* **98**, 254–267 (2014).

10. W. K. Hartmann, A. Morbidelli, Effects of early intense bombardment on megaregolith evolution and on lunar (and planetary) surface samples. *Meteorit. Planet. Sci.* **55**, 2472–2492 (2020).

11. A. Morbidelli, S. Marchi, W. F. Bottke, D. A. Kring, A sawtooth-like timeline for the first billion years of lunar bombardment. *Earth Planet. Sci. Lett.* **355-356**, 144–151 (2012).

12. M. D. Hopkins, S. J. Mojzsis, A protracted timeline for lunar bombardment from mineral chemistry, Ti thermometry and U–Pb geochronology of Apollo 14 melt breccia zircons. *Contrib. Mineral. Petrol.* **169**, 30 (2015).

13. G. Neukum, B. A. Ivanov, W. K. Hartmann, Cratering records in the inner solar system in relation to the lunar reference system. *Space Sci. Rev.* **96**, 55–86 (2001).

14. J. Y. Chen, L. Zhang, Z. X. Cui, Z. M. Chen, Z. Y. Xiao, F. L. Luo, Y. Q. Qian, Y. Q. Zhang, C. Y. Wang, J. T. Wang, Q. Yang, P. L. He, L. L. Chen, F. F. Huang, H. Y. Xian, K. H. Joy, J. W. Head, C. R. Neal, Y. G. Xu, KREEP-like lithologies in the South Pole-Aitken basin reworked by the Apollo basin impact at 4.16 Ga. *Nat. Astron.* **9**, 1638–1647 (2025).

15. Q. Zhou, W. Yang, Z. Y. Chu, H. G. Zhu, S. H. Yang, X. G. Zeng, D. S. Xue, L. H. Jia, G. L. Zhang, H. B. Zhang, Y. H. Lin, H. J. Zhang, H. C. Tian, P. Peng, D. P. Zhang, L. X. Gu, C. L. Li, F. Y. Wu, Ultra-depleted mantle source of basalts from the South Pole–Aitken basin. *Nature* **643**, 371–375 (2025).

16. A. Morbidelli, D. Nesvorny, V. Laurenz, S. Marchi, D. C. Rubie, L. Elkins-Tanton, M. Wieczorek, S. Jacobson, The timeline of the lunar bombardment: Revisited. *Icarus* **305**, 262–276 (2018).

17. G. B. Dalrymple, G. Ryder, $^{40}Ar/^{39}Ar$ age spectra of Apollo-15 impact melt rocks by laser step-heating and their bearing on the history of lunar basin formation. *J. Geophys. Res. Planets* **98**, 13085–13095 (1993).

18. G. B. Dalrymple, G. Ryder, $^{40}Ar/^{39}Ar$ age spectra of Apollo 17 highlands breccia samples by laser step heating and the age of the Serenitatis Basin. *J. Geophys. Res.* **101**, 26069–26084 (1996).

19. P. H. Cadogan, G. Turner, $^{40}Ar/^{39}Ar$ dating of luna 16 and luna 20 samples. *Philos. Trans. A Math Phys. Eng. Sci.* **284**, 167–177 (1977).

20. R. Gomes, H. F. Levison, K. Tsiganis, A. Morbidelli, Origin of the cataclysmic Late Heavy Bombardment period of the terrestrial planets. *Nature* **435**, 466–469 (2005).

21. K. Tsiganis, R. Gomes, A. Morbidelli, H. F. Levison, Origin of the orbital architecture of the giant planets of the Solar System. *Nature* **435**, 459–461 (2005).

22. W. K. Hartmann, Lunar "cataclysm": A misconception. *Icarus* **24**, 181–187 (1975).

23. W. K. Hartmann, History of the terminal cataclysm paradigm; epistemology of a planetary bombardment that never (?) happened. *Geosciences* **9**, 285 (2019).

24. Z. Y. Yue, S. Gou, Y. X. Wang, H. C. Li, G. Michael, J. Z. Liu, S. J. Sun, Y. T. Lin, K. C. Di, Q. L. Li, Y. Chen, W. Yang, B. Xie, S. Hu, S. D. Li, B. Zheng, T. Q. Mao, X. H. Li, F. Y. Wu, Lunar chronology model with the Chang'e-6 farside samples and implications for the early impact history. *Sci. Adv.* **12**, eady9265 (2026).

25. N. E. B. Zellner, Cataclysm no more: New views on the timing and delivery of lunar impactors. *Orig. Life Evol. Biosph.* **47**, 261–280 (2017).

26. R. Tartèse, M. Anand, J. Gattacceca, K. H. Joy, J. I. Mortimer, J. F. Pernet-Fisher, S. Russell, J. F. Snape, B. P. Weiss, Constraining the evolutionary history of the moon and the inner solar system: A case for new returned lunar samples. *Space Sci. Rev.* **215**, 54 (2019).

27. A. A. Nemchin, T. Long, B. L. Jolliff, Y. Wan, J. F. Snape, R. Zeigler, M. L. Grange, D. Liu, M. J. Whitehouse, N. E. Timms, F. Jourdan, Ages of lunar impact breccias: Limits for timing of the Imbrium impact. *Geochemistry* **81**, 125683 (2021).

28. D. Y. Liu, B. L. Jolliff, R. A. Zeigler, R. L. Korotev, Y. S. Wan, H. Q. Xie, Y. H. Zhang, C. Y. Dong, W. Wang, Comparative zircon U-Pb geochronology of impact melt breccias from Apollo 12 and lunar meteorite SaU 169, and implications for the age of the Imbrium impact. *Earth Planet. Sci. Lett.* **319**, 277–286 (2012).

29. R. E. Merle, A. A. Nemchin, M. L. Grange, M. J. Whitehouse, R. T. Pidgeon, High resolution U-Pb ages of Ca-phosphates in Apollo 14 breccias: Implications for the age of the Imbrium impact. *Meteorit. Planet. Sci.* **49**, 2241–2251 (2014).

30. C. M. Mercer, K. E. Young, J. R. Weirich, K. V. Hodges, B. L. Jolliff, J. Wartho, M. C. van Soest, Refining lunar impact chronology through high spatial resolution ^{40}Ar/^{39}Ar dating of impact melts. *Sci. Adv.* **1**, e1400050 (2015).

31. T. M. Harrison, B. Zhang, A. F. Parisi, E. A. Bell, A failed search for concordancy across multiple isotopic systems in lunar impactites: Implications for testing the Late Heavy Bombardment hypothesis. *Earth Planet. Sci. Lett.* **646**, 118943 (2024).

32. G. Michael, A. Basilevsky, G. Neukum, On the history of the early meteoritic bombardment of the Moon: Was there a terminal lunar cataclysm? *Icarus* **302**, 80–103 (2018).

33. B. A. Cohen, T. D. Swindle, D. A. Kring, Support for the lunar cataclysm hypothesis from lunar meteorite impact melt ages. *Science* **290**, 1754–1756 (2000).

34. B. A. Cohen, T. D. Swindle, D. A. Kring, Geochemistry and ^{40}Ar-^{39}Ar geochronology of impact-melt clasts in feldspathic lunar meteorites: Implications for lunar bombardment history. *Meteorit. Planet. Sci.* **40**, 755–777 (2005).

35. B. Su, Y. Chen, Z. L. Wang, D. Zhang, H. J. Chen, S. Gou, Z. Y. Yue, Y. H. Liu, J. Y. Yuan, G. Q. Tang, S. Guo, Q. Li, Y. T. Lin, X. H. Li, F. Y. Wu, South Pole-Aitken massive impact 4.25 billion years ago revealed by Chang'e-6 samples. *Natl. Sci. Rev.* **12**, nwaf103 (2025).

36. K. H. Joy, N. Wang, J. F. Snape, A. Goodwin, J. F. Pernet-Fisher, M. J. Whitehouse, Y. Liu, Y. T. Lin, J. R. Darling, P. Tar, R. Tartèse, Evidence of a 4.33 billion year age for the Moon's South Pole-Aitken basin. *Nat. Astron.* **9**, 55–65 (2025).

37. J. Y. Chen, S. L. Li, S. Y. Liao, J. Chen, A. Nemchin, K. H. Joy, X. C. Che, W. B. Hsu, M. H. Zhu, Reprocessing of lunar crust at □4.3 Ga inferred from in situ U-Pb isotopic and trace element investigation of Northwest Africa 11479. *Geochim. Cosmochim. Acta* **415**, 56–70 (2026).

38. J. A. Cartwright, K. V. Hodges, M. Wadhwa, Evidence against a Late Heavy Bombardment event on Vesta. *Earth Planet. Sci. Lett.* **590**, 117576 (2022).

39. L. F. White, D. E. Moser, J. R. Darling, B. G. Rider-Stokes, B. Hyde, K. T. Tait, K. Chamberlain, A. K. Schmitt, J. Dunlop, M. Anand, Accessory mineral microstructure and chronology reveals no evidence for late heavy bombardment on the asteroid 4-Vesta. *Earth Planet. Sci. Lett.* **636**, 118694 (2024).

40. F. Jourdan, T. Kennedy, L. Forman, C. Mayers, E. Eroglu, A. Yamaguchi, A slowly cooled deep crust on asteroid 4 Vesta and the recent impact history of rubble pile vestoids recorded by diogenites. *Geochim. Cosmochim. Acta* **365**, 35–52 (2024).

41. P. Boehnke, T. M. Harrison, Illusory late heavy bombardments. *Proc. Natl. Acad. Sci. U.S.A.* **113**, 10802–10806 (2016).

42. D. D. Bogard, K–Ar ages of meteorites: Clues to parent-body thermal histories. *Geochemistry* **71**, 207–226 (2011).

43. C. L. Li, H. Hu, M. F. Yang, J. J. Liu, Q. Zhou, X. Ren, B. Liu, D. W. Liu, X. G. Zeng, W. Zuo, G. L. Zhang, H. B. Zhang, S. H. Yang, Q. Wang, X. J. Deng, X. Y. Gao, Y. Su, W. B. Wen, Z. Y. Ouyang, Nature of the lunar farside samples returned by the Chang'E-6 mission. *Natl. Sci. Rev.* **11**, nwae328 (2024).

44. I. Garrick-Bethell, M. T. Zuber, Elliptical structure of the lunar South Pole-Aitken basin. *Icarus* **204**, 399–408 (2009).

45. G. A. Neumann, M. T. Zuber, M. A. Wieczorek, J. W. Head, D. M. H. Baker, S. C. Solomon, D. E. Smith, F. G. Lemoine, E. Mazarico, T. J. Sabaka, S. J. Goossens, H. J. Melosh, R. J. Phillips, S. W. Asmar, A. S. Konopliv, J. G. Williams, M. M. Sori, J. M. Soderblom, K. Miljković, J. C. Andrews-Hanna, F. Nimmo, W. S. Kiefer, Lunar impact basins revealed by Gravity Recovery and Interior Laboratory measurements. *Sci. Adv.* **1**, e1500852 (2015).

46. P. D. Spudis, J. J. Gillis, R. A. Reisse, Ancient multiring basins on the moon revealed by Clementine laser altimetry. *Science* **266**, 1848–1851 (1994).

47. Z. M. Chen, L. Zhang, J. Y. Chen, C. Y. Wang, J. T. Wang, Z. X. Cui, Z. Y. Zou, P. L. He, Y. H. Cao, Q. Zhou, L. L. Chen, Y. Q. Zhang, Y.-G. Xu, Abundant non-mare components in the

Chang’e-6 lunar regolith: Constraints from plagioclase fragments and impact glasses. *J. Geophys. Res. Planets* **130**, e2025JE008976 (2025).

48. M. D. Norman, R. A. Duncan, J. J. Huard, Identifying impact events within the lunar cataclysm from ^{40}Ar-^{39}Ar ages and compositions of Apollo 16 impact melt rocks. *Geochim. Cosmochim. Acta* **70**, 6032–6049 (2006).

49. W. F. Zhang, J. C. Wu, J. J. Wang, L. Zhang, Y. G. Xu, Y. Q. Zhang, P. L. He, D. W. Zheng, F. Su, H. Y. He, M. Xiao, Y. D. Jiang, F. F. Huang, J. Z. Wang, Y. H. Cao, L. L. Chen, A novel ultralow-consumption technique for mafic rock major and trace element analysis. *Anal. Chem.* **97**, 24058–24064 (2025).

50. P. A. Baedecker, J. T. Wasson, Gallium, germanium, indium, and iridium in lunar samples. *Science* **167**, 503–505 (1970).

51. R. L. Korotev, The nature of the meteoritic components of Apollo 16 soil, as inferred from correlations of iron, cobalt, iridium, and gold with nickel. *J. Geophys. Res.* **92**, E447–E461 (1987).

52. T. C. Labotka, M. J. Kempa, C. White, J. J. Papike, J. C. Laul, “The lunar regolith: Comparative petrology of the Apollo sites,” in *11th Lunar and Planetary Science Conference* (Houston, 1980), pp. 1285–1305.

53. F. Jourdan, T. Kennedy, G. K. Benedix, E. Eroglu, C. Mayer, Timing of the magmatic activity and upper crustal cooling of differentiated asteroid 4 Vesta. *Geochim. Cosmochim. Acta* **273**, 205–225 (2020).

54. S. Mighani, H. Wang, D. L. Shuster, C. S. Borlina, C. I. O. Nichols, B. P. Weiss, The end of the lunar dynamo. *Sci. Adv.* **6**, eaax0883 (2020).

55. J. P. Das, S. L. Baldwin, J. W. Delano, ^{40}Ar/^{39}Ar and cosmic ray exposure ages of plagioclase-rich lithic fragments from Apollo 17 regolith, 78461. *Earth Planets Space* **68**, e14890 (2016).

56. Z. X. Cui, Q. Yang, Y. Q. Zhang, C. Y. Wang, H. Y. Xian, Z. M. Chen, Z. Y. Xiao, Y. Q. Qian, J. W. Head, C. R. Neal, L. Xiao, F. L. Luo, J. Y. Chen, P. L. He, Y. H. Cao, Q. Zhou, F. F.

Huang, L. L. Chen, B. Wei, J. T. Wang, Y. N. Yang, S. Li, Y. P. Yang, X. J. Lin, J. X. Zhu, L. Zhang, Y. G. Xu, A sample of the Moon's far side retrieved by Chang'e-6 contains 2.83-billion-year-old basalt. *Science* **386**, 1395–1399 (2024).

57. X. C. Che, T. Long, A. Nemchin, S. W. Xie, L. Qiao, Z. S. Li, Y. Y. Ban, R. L. Fan, C. Yang, D. Y. Liu, Isotopic and compositional constraints on the source of basalt collected from the lunar far side. *Science* **387**, 1306–1310 (2025).

58. Q. W. L. Zhang, M. H. Yang, Q. L. Li, Y. Liu, Z. Y. Yue, Q. Zhou, L. Y. Chen, H. X. Ma, S. H. Yang, X. Tang, G. L. Zhang, X. Ren, X. H. Li, Lunar farside volcanism 2.8 billion years ago from Chang'e-6 basalts. *Nature* **643**, 356–360 (2025).

59. B. B. Hanan, G. R. Tilton, 60025: Relict of primitive lunar crust? *Earth Planet. Sci. Lett.* **84**, 15–21 (1987).

60. L. E. Borg, J. N. Connelly, M. Boyet, R. W. Carlson, Chronological evidence that the Moon is either young or did not have a global magma ocean. *Nature* **477**, 70–72 (2011).

61. Z. Y. Yue, S. Gou, S. J. Sun, W. Yang, Y. Chen, Y. X. Wang, H. L. Lin, K. C. Di, Y. T. Lin, X. H. Li, F. Y. Wu, Geological context of the Chang'e-6 landing area and implications for sample analysis. *Innovation* **5**, 100663 (2024).

62. F. Jourdan, N. E. Timms, T. Nakamura, W. D. A. Rickard, C. Mayers, S. M. Reddy, D. Saxey, L. Daly, P. A. Bland, E. Eroglu, D. Fougerouse, Rubble pile asteroids are forever. *Proc. Natl. Acad. Sci. U.S.A.* **120**, e2214353120 (2023).

63. F. Jourdan, The $^{40}Ar/^{39}Ar$ dating technique applied to planetary sciences and terrestrial impacts. *Aust. J. Earth Sci.* **59**, 199–224 (2012).

64. L. Y. Xu, L. Qiao, M. G. Xie, Y. R. Wang, M. H. Zhu, J. G. Yan, Chronology, local stratigraphy, and foreign ejecta materials at the Chang'e-6 landing site: Constraints on the provenance of samples returned from the Moon's farside. *Geophys. Res. Lett.* **51**, e2024GL111311 (2024).

65. D. M. Chew, R. A. Spikings, Geochronology and thermochronology using apatite; time and temperature, lower crust to surface. *Elements* **11**, 189–194 (2015).

66. C. Orgel, G. Michael, C. I. Fassett, C. H. van der Bogert, C. Riedel, T. Kneissl, H. Hiesinger, Ancient bombardment of the inner solar system: Reinvestigation of the "fingerprints" of different impactor populations on the lunar surface. *J. Geophys. Res. Planets* **123**, 748–762 (2018).

67. J. Y. Zhang, J. Z. Liu, Thorium anomaly on the lunar surface and its indicative meaning. *Acta Geochim.* **43**, 507–519 (2024).

68. R. L. Korotev, R. A. Zeigler, B. L. Jolliff, A. J. Irvin, T. E. Bunch, Compositional and lithological diversity among brecciated lunar meteorites of intermediate iron concentration. *Meteorit. Planet. Sci.* **44**, 1287–1322 (2009).

69. Y. G. Xu, J. Y. Chen, The oldest anchor for lunar crater chronology constrained by the new age of 4.25 Ga of the South Pole-Aitken basin. *Natl. Sci. Rev.* **12**, nwaf164 (2025).

70. L. A. Haskin, R. L. Korotev, K. M. Rockow, B. L. Jolliff, The case for an Imbrium origin of the Apollo thorium-rich impact-melt breccias. *Meteorit. Planet. Sci.* **33**, 959–975 (1998).

71. J. R. Head, C. I. Fassett, S. J. Kadish, D. E. Smith, M. T. Zuber, G. A. Neumann, E. Mazarico, Global distribution of large lunar craters: Implications for resurfacing and impactor populations. *Science* **329**, 1504–1507 (2010).

72. K. Miljkovicc, M. A. Wieczorek, G. S. Collins, M. Laneuville, G. A. Neumann, H. J. Melosh, S. C. Solomon, R. J. Phillips, D. E. Smith, M. T. Zuber, Asymmetric distribution of lunar impact basins caused by variations in target properties. *Science* **342**, 724–726 (2013).

73. W. F. Zhang, D. W. Zheng, J. Fred, F. Adam, M. Celia, Y. G. Xu, H. Y. He, Y. Q. Zhang, J. J. Wang, Y. D. Jiang, M. Xiao, J. J. Li, J. Zhang, ZMT04 muscovite: A potential Paleoproterozoic reference material for $^{40}Ar/^{39}Ar$ dating. *J. Anal. At. Spectrom* **39**, 2173–2182 (2024).

74. P. R. Renne, G. Balco, K. R. Ludwig, R. Mundil, K. Min, Response to the comment by W.H. Schwarz et al. on "Joint determination of ^{40}K decay constants and ^{40}Ar*/^{40}K for the Fish Canyon sanidine standard, and improved accuracy for ^{40}Ar/^{39}Ar geochronology" by P. R. Renne et al. (2010). *Geochim. Cosmochim. Acta* **75**, 5097–5100 (2011).

75. F. Jourdan, A. Frew, A. Joly, C. Mayers, N. J. Evans, WA1ms: A □2.61 Ga muscovite standard for ^{40}Ar/^{39}Ar dating. *Geochim. Cosmochim. Acta* **141**, 113–126 (2014).

76. W. F. Zhang, J. J. Wang, D. W. Zheng, M. Xiao, J. Zhang, J. J. Li, Y. L. Zhang, Y. D. Jiang, F. Su, L. Li, Y. G. Xu, Correction for calcium interference in the ^{40}Ar/^{39}Ar dating method. *Icarus* **435**, 116591 (2025).

77. J. Y. Lee, K. Marti, J. P. Severinghaus, K. Kawamura, H. S. Yoo, J. B. Lee, J. S. Kim, A redetermination of the isotopic abundances of atmospheric Ar. *Geochim. Cosmochim. Acta* **70**, 4507–4512 (2006).

78. R. Wieler, Noble gases in the solar system. *Rev. Mineral. Geochem.* **47**, 21–70 (2002).

79. P. Eberhardt, J. Geiss, H. Graf, N. Grögler, M. D. Mendia, M. Mörgeli, H. Schwaller, A. Stettler, U. Krähenbühl, H. R. von Gunten, Trapped solar wind noble gases in Apollo 12 lunar fines 12001 and Apollo 11 breccia 10046. *Proc. Lunar Sci. Conf.* **3**, 1821–1856 (1972).

80. M. D. Norman, F. Jourdan, S. S. M. Hui, Impact history and regolith evolution on the moon: Geochemistry and ages of glasses from the Apollo 16 site. *J. Geophys. Res. Planets* **124**, 3167–3180 (2019).

81. T. S. Culler, T. A. Becker, R. A. Muller, P. R. Renne, Lunar impact history from ^{40}Ar/^{39}Ar dating of glass spherules. *Science* **287**, 1785–1788 (2000).

82. F. Su, X. H. Zhang, Y. J. Li, B. R. Jicha, K. H. Joy, Q. L. Li, Y. Chen, S. H. Cai, R. C. Liu, Q. Zhou, S. H. Yang, X. H. Li, L. K. Yang, W. Chen, J. J. Li, W. F. Zhang, H. N. Qiu, H. Y. He, Constraining 2.0 Ga volcanism on the moon via ^{40}Ar/^{39}Ar dating of Chang'e-5 basalts. *J. Geophys. Res. Planets* **130**, e2024JE008495 (2025).

83. A. A. P. Koppers, ArArCALC - Software for $^{40}Ar/^{39}Ar$ age calculations. *Comput. Geosci.* **28**, 605–619 (2002).

84. L. Zhang, Z. Y. Ren, X. P. Xia, Q. Yang, L. B. Hong, D. Wu, In situ determination of trace elements in melt inclusions using laser ablation inductively coupled plasma sector field mass spectrometry. *Rapid Commun. Mass Spectrom.* **33**, 361–370 (2019).

85. L. J. Hallis, M. Anand, S. Strekopytov, Trace-element modelling of mare basalt parental melts: Implications for a heterogeneous lunar mantle. *Geochim. Cosmochim. Acta* **134**, 289–316 (2014).

86. H. Wiesmann, N. Hubbard, "A compilation of the lunar sample data generated by the Gast, Nyquist, and Hubbard lunar sample PI-ships" (NASA Johnson Space Center, 1975).

87. L. A. Haskin, M. M. Lindstrom, P. A. Salpas, D. J. Lindstrom, "On compositional variations among lunar anorthosites," in *Proceedings 12th Lunar and Planetary Science Conference* (LPSC, 1981) , pp. 41–66.

88. W. I. Ridley, N. J. Hubbard, J. M. Rhodes, H. Weismann, B. Bansal, Petrology of Lunar breccia - 15445 and petrogenetic implications. *J. Geol.* **81**, 621–631 (1973).
